# Development and validation of a hypoxia- and mitochondrial dysfunction- related prognostic model based on integrated single-cell and bulk RNA sequencing analyses in gastric cancer

**DOI:** 10.3389/fimmu.2024.1419133

**Published:** 2024-08-06

**Authors:** Yirong Li, Yue Cui, Zhen Wang, Liwei Wang, Yi Yu, Yuyan Xiong

**Affiliations:** ^1^ Xi’an Key Laboratory of Cardiovascular and Cerebrovascular Diseases, Xi’an No.3 Hospital, The Affiliated Hospital of Northwest University, Xi’an, Shaanxi, China; ^2^ Key Laboratory of Resource Biology and Biotechnology in Western China, Ministry of Education, College of Life Sciences, Northwest University, Xi’an, Shaanxi, China; ^3^ School of Medicine, Northwest University, Xi’an, Shaanxi, China

**Keywords:** gastric cancer, hypoxia, mitochondrial dysfunction, prognosis, model, immunotherapy

## Abstract

**Introduction:**

Gastric cancer (GC) remains a major global health threat ranking as the fifth most prevalent cancer. Hypoxia, a characteristic feature of solid tumors, significantly contributes to the malignant progression of GC. Mitochondria are the major target of hypoxic injury that promotes mitochondrial dysfunction during the development of cancers including GC. However, the gene signature and prognostic model based on hypoxia- and mitochondrial dysfunction-related genes (HMDRGs) in the prediction of GC prognosis have not yet been established.

**Methods:**

The gene expression profile datasets of stomach cancer patients were retrieved from The Cancer Genome Atlas and the Gene Expression Omnibus databases. Prognostic genes were selected using Least Absolute Shrinkage and Selection Operator Cox (LASSO-Cox) regression analysis to construct a prognostic model. Immune infiltration was evaluated through ESTIMATE, CIBERSORT, and ssGSEA analyses. Tumor immune dysfunction and exclusion (TIDE) and immunophenoscore (IPS) were utilized to explore implications for immunotherapy. Furthermore, in vitro experiments were conducted to validate the functional roles of HMDRGs in GC cell malignancy.

**Results:**

In this study, five HMDRGs (ZFP36, SERPINE1, DUSP1, CAV1, and AKAP12) were identified for developing a prognostic model in GC. This model stratifies GC patients into high- and low-risk groups based on median risk scores. A nomogram predicting overall survival (OS) was constructed and showed consistent results with observed OS. Immune infiltration analysis indicated that individuals in the high-risk group tend to exhibit increased immune cell infiltration. Additionally, analysis of cancer immunotherapy responses revealed that high-risk group patients exhibit poorer responses to cancer immunotherapy compared to the low-risk group. Immunohistochemistry (IHC) staining indicated that the expression levels of HMDRGs were remarkably correlated with GC, of which, SERPINE1 displayed the most pronounced up-regulation, while ZFP36 exhibited the most notable down-regulation in GC patients. Furthermore, *in vitro* investigation validated that SERPINE1 and ZFP36 contribute to the malignant processes of GC cells correlated with mitochondrial dysfunction.

**Conclusions:**

This study presents a novel and efficient approach to evaluate GC prognosis and immunotherapy efficacy, and also provides insights into understanding the pathogenesis of GC.

## 1 Introduction

Gastric cancer (GC) is a common malignancy, ranking as the third leading cause of global cancer-related mortality (1). Despite eminent advancements achieved in diagnosing and therapeutic interventions, the survival rate for GC remains unsatisfactory all over the world, which is partially attributed to be lack of efficient tools to predict prognosis, identify high-risk patients, and assess immunotherapy responses (2, 3). This underscores the urgent need for exploring novel approaches to tackle this critical problem. In recent years, developing a prognostic model based on gene signatures offers a promising strategy.

Hypoxia, one of the common cellular stresses, is a distinctive feature observed in solid tumors, and plays multiple roles in tumor biology through the modulation of tumor cell proliferation and migration, immune evasion, angiogenesis, microenvironment, invasion, and metastasis (4). Over the past decades, emerging studies have demonstrated that hypoxia contributes to GC malignant progression (5–7). Hypoxia-inducible factor-1α (HIF-1α), a key modulator under hypoxia conditions, has been clinically revealed that its positive expression is significantly correlated with GC progression and development, and it can be a potent inducer in GC (8, 9). Moreover, HIF-1α has been demonstrated to be an effective biomarker for predicting the outcomes of GC patients in a predictive model based on three ferroptosis‐related genes including HIF-1α, cation transport regulator homolog 1 (CHAC1) and NADPH oxidase 4 (NOX4) (10). Notably, long-term hypoxia can cause severe damage to mitochondria to induce mitochondrial dysfunction by producing excessive reactive oxygen species (ROS) (11), and disrupting biogenesis, fission, and morphology (12). Mitochondria is the energy production center of cells, and its dysfunction not only dysregulates cellular energy metabolism but also triggers a series of disruptions in cellular signaling pathways, in turn exacerbating cancer development (13). Substantial evidence has indicated that both hypoxia and mitochondrial dysfunction play a central role in contributing to the malignant progression of GC (14–16), and are widely acknowledged to correlate with poorer prognosis in GC (17, 18). Whereas, a predictive prognosis model based on hypoxia- and mitochondrial dysfunction-related genes (HMDRGs) has yet to be established.

In this study, we identified five HMDRGs through a comprehensive bioinformatics analysis. A prognostic model based on this HMDRG signature was established, and its prognostic value was validated in GC patient cohorts. A nomogram model for predicting overall survival (OS) was also constructed, exhibiting consistent values with the actual observed OS. More importantly, we validated the biological functions and potential molecular mechanisms of HMDRGs in contributing to the malignant processes in GC cell lines via modulating mitochondrial dysfunction. This study may open up new avenues for clinical prognostic prediction, risk stratification and evaluation of immunotherapy response in GC. Furthermore, we provide novel insights into understanding the molecular mechanisms of GC pathogenesis connecting to hypoxia and mitochondrial dysfunction.

## 2 Materials and methods

### 2.1 Acquisition of hypoxia- and mitochondrial dysfunction-related gene sets

The hypoxia-related gene set consisting of 200 genes was retrieved from the Molecular Signatures Database (http://www.gsea-msigdb.org/gsea/msigdb/index.jsp) (**Supplementary Table S1**). The human mitochondrial dysfunction-related gene set was obtained from the GeneCards database (https://www.genecards.org/) using the keyword search term “mitochondrial dysfunction” and a relevance score threshold of >1.5 (**Supplementary Table S2**).

### 2.2 Data collection

The RNA-seq data and clinical characteristics of the TCGA STAD cohorts for training purposes were collected from the TCGA database (https://portal.gdc.cancer.gov/). Participants lacking detailed expression and clinical data or with a 0-day follow-up duration were excluded. Following these criteria, 348 STAD and 32 normal samples were obtained and selected for the training cohorts (19). To validate our model, GSE84437 and GSE62254 STAD cohorts were retrieved from the Gene Expression Omnibus (GEO) public repository (https://www.ncbi.nlm.nih.gov/geo/). Log2 transformation and normalization were applied to the expression profiles, and the average expression level was used for duplicate genes. The “ComBat” function of the “sva” package (v3.50.0) (https://bioconductor.org/packages/release/bioc/html/sva.html) in R software version 4.2.1 was utilized to remove batch effects (20). Ten stomach tumor tissue samples from patients with primary gastric cancer were utilized for single-cell RNA sequencing analysis, obtained from dataset GSE183904 in the GEO database (https://www.ncbi.nlm.nih.gov/geo/).

### 2.3 scRNA-seq data analysis

The GC single-cell RNA sequencing (scRNA-seq) data underwent analysis utilizing the “Seurat” R package (v4.3.0) (21, 22). Initial data are screened according to the criteria of cells with less than 15% mitochondrial genes, 200 to 7000 genes per cell, and each gene expressed in at least three cells, ensuring high-quality scRNA-seq data. To exclude batch effects and integrate diverse single-cell transcriptome samples, the “SCTransform” function within the “Seurat” package was employed. Subsequently, highly variable genes were selected via the “Select Integration Features” function for anchoring purposes. Dimensionality reduction was executed using the “RunPCA” function, with a specified dimension of 50. Cluster analysis was conducted with the “Find Clusters” program, setting the resolution parameter at 0.5. T-distributed Stochastic Neighbor Embedding (t-SNE) was employed to compress high-dimensional probability distributions into a lower-dimensional space.

### 2.4 Identification of single-cell hypoxia-related differentially expressed genes

To evaluate the degree of enrichment for hypoxia-related gene expression at the single-cell level, we executed the “AUCell” R package (v1.24.0) (23). We computed the hypoxia AUC value for each cell type using 200 hypoxia-related genes. The cells were stratified into high hypoxia-AUC score and low hypoxia-AUC score groups based on the median AUC score as the cutoff value. The partitioning visualization was performed using the “FindMarkers” function of Seurat R package. Genes were identified as single-cell hypoxia-related differentially expressed genes (DEGs) according to the criteria of an adjusted p-value< 0.05, |log2FoldChange| > 1, and Minpct ≥ 0.25.

### 2.5 Identification of differentially expressed genes of STAD

The “edgeR” package (v4.0.16) was utilized for conducting differential expression analysis of genes between TCGA STAD and normal stomach tissue. Genes with adjusted p-value (adj.p)< 0.05 and |log2FoldChange| > 1 were identified as differentially expressed genes (DEGs) of STAD (24).

### 2.6 Identification and validation of the prognostic HMDRG gene signature

Univariate Cox proportional hazards regression analysis was conducted on each HMDRG to identify genes significantly associated with OS in the TCGA training cohort (25, 26). Subsequently, the LASSO Cox regression method was applied to further identify HMDRGs using the R software package “glmnet” (version 4.1-8) (27, 28). Based on the optimal lambda value, HMDRGs were identified and used to calculate a prognostic risk score for each patient using the following formula:


$$Risk\;Score=\sum^{\;}expr_{i}\;*\;coef_{i}$$

where “coef” represents the regression coefficients of each HMDRG, and “expr” denotes the expression values. The median risk score was defined as the cutoff value to divide TCGA STAD patients into high-risk and low-risk groups. Univariate and multivariate Cox proportional hazards regression analyses were conducted to assess whether the HMDRG-based prognostic model was an independent prognostic factor combined with clinical variables. A Kaplan-Meier (K-M) survival curve was generated, and survival differences between groups were assessed using the log-rank test. The sensitivity and specificity of the prognostic performance were evaluated through ROC curve analysis and visualized using the R package “timeROC” (v0.4) (29). The area under the curve (AUC) values indicated the discrimination ability of the model.

### 2.7 The development and validation of the nomogram

A prognostic nomogram was constructed using the “rms” package (v6.8.0) in R software to estimate the probability of 1-, 3-, and 5-year overall survival (OS) in STAD patients. The calibration curves and the Concordance Index (C-Index) were employed to assess the predictive accuracy of our nomogram. Calibration curves were used to visually compare the predicted probabilities generated by our model with the actual observed survival rates, offering valuable insights into the precision and reliability of predicting patient prognoses. Furthermore, we utilized the C-Index to assess the predictive accuracy. Calculating the C-Index AUC value allowed us to quantitatively assess the predictive capabilities of the model.

### 2.8 Gene set enrichment analysis

GSEA was conducted using the R package “clusterProfiler” (v4.10.1) to explore biological pathways associated with the high-risk and low-risk groups in stomach cancer patients from TCGA (30). The analysis ranked the gene list based on their signal-to-noise ratio and used a reference database of known pathways (c2.cp.kegg.v7.5.1.entrez.gmt). Pathways with a normalized enrichment score (|NES|) greater than 1 and a p-value less than 0.05 were considered significantly enriched (31).

### 2.9 Functional enrichment analysis

To elucidate the biological significance of the HMDRGs, functional enrichment analysis was performed using the ‘clusterProfiler’ package (v4.10.1) in R (32, 33), including Gene Ontology (GO) and Kyoto Encyclopedia of Genes and Genomes (KEGG) pathway enrichment to characterize the functional profiles of the HMDRGs. The p-value less than 0.05 was defined as statistically significant enrichment.

### 2.10 Immune landscape analysis

To characterize the immune landscape of gastric cancer, gene expression profiles from tumor samples were used to estimate the proportions of immune and stromal cells within the tumor microenvironment (TME) for each patient. The “ESTIMATE” R package (v1.0.13) was employed to calculate the stromal score (indicating the presence of supportive tissue), immune score (reflecting the extent of immune cell infiltration), ESTIMATE score (combined stromal and immune scores), and tumor purity. CIBERSORT analysis was used to deconvolute the cellular composition of the tumor sample of each STAD patient based on expression profiles, which identified the relative abundances of 22 distinct immune cell types (34). The single-sample gene set enrichment analysis (ssGSEA) implemented within the “GSVA” R package (v1.50.5) was employed to estimate the infiltration levels of 28 different immune cell types (35, 36).

### 2.11 Immunotherapy responses analysis

The analyses of tumor immune dysfunction and exclusion (TIDE) score and immunophenoscore (IPS) were employed to explore the potential of our model in cancer immunotherapy. TIDE is a computational tool used to assess tumor immune evasion and predict response to immune checkpoint inhibitors (ICIs) therapy by integrating multiple biomarkers to analyze interactions between the tumor and immune system, and its score obtained from the Harvard TIDE website (http://tide.dfci.harvard.edu/) (37). IPS analysis is an algorithm used to predict the potential response to cancer immunotherapy by integrating multiple gene expression profiles and immune-related biomarkers to provide a comprehensive assessment of the tumor immune landscape, and obtained from the Cancer Imaging Archive (TCIA) database (https://tcia.at/home) (38–41).

### 2.12 Immunohistochemical staining analysis of HMDRGs protein in GC samples

Immunohistochemical staining analysis of HMDRGs protein expression levels was employed by accessing the immunohistochemical staining images of HMDRGs protein in GC pathological sections from the Human Protein Atlas (HPA) database (https://www.proteinatlas.org/).

### 2.13 The potential drug prediction

To identify potential therapeutic targets, we performed gene-based drug screening using data from DrugBank (https://go.drugbank.com). The Protein Data Bank (PDB) database (https://www.rcsb.org) was queried to retrieve the crystal structures of relevant drug target proteins. We employed Autodock 4 software (v4.0) for in silico molecular docking simulations to investigate the binding interactions between the identified drugs and their corresponding target proteins.

### 2.14 Cell culture

MGC803, HGC27 human gastric cancer cells were purchased from Procell in Wuhan, China, and cultured in DMEM medium (DMEM; Sigma, USA) supplemented with 10% fetal bovine serum (Gemini, USA), 100 g/mL streptomycin, and 100 U/mL penicillin at 37 °C. For hypoxic treatment, cells were incubated in an incubator with 1% O_2_ and 94% N_2_, while normoxic conditions were maintained in an incubator with 21% O_2_ and 5% CO_2_. Forskolin treatment was performed at concentrations of 20 μM and 40 μM.

### 2.15 Quantitative real-time PCR

Total RNA was isolated from cells using TRIzol reagent (Invitrogen) according to the manufacturer’s protocol. Reverse transcription was performed with the PrimeScript RT Reagent Kit (Takara) following the manufacturer’s instructions. Quantitative reverse transcription-polymerase chain reaction (RT-qPCR) was then performed using the SYBR PrimeScript RT-PCR Kit (Takara) to analyze gene expression. The 2^-ΔΔCT^ method was employed for quantification, with β-actin used as an internal control. The PCR primer pairs, synthesized by Sangon Biotech in Shanghai, China, had the following sequences (5’-3’, F: forward, R: reverse, h: human):

hZFP36-F: GCTATGTCGGACCTTCTCAGAG,

hZFP36-R: CCTGGAGGTAGAACTTGTGACAG;

hSERPINE1-F: CTCATCAGCCACTGGAAAGGCA,

hSERPINE1-R: GACTCGTGAAGTCAGCCTGAAAC;

hDUSP1-F: CAACCACAAGGCAGACATCAGC,

hDUSP1-R: GTAAGCAAGGCAGATGGTGGCT;

hCAV1-F: CCAAGGAGATCGACCTGGTCAA,

hCAV1-R: GCCGTCAAAACTGTGTGTCCCT;

hAKAP12-F: AGAAAGGAGCCCTGAACGGTCA,

hAKAP12-R: CCGCTGACTTAGTAGCCATCTC;

hβ-actin -F: CACCATTGGCAATGAGCGGTTC,

hβ-actin -R: AGGTCTTTGCGGATGTCCACGT.

### 2.16 Cell proliferation assay

To evaluate cell proliferation, the Cell Counting Kit-8 (CCK-8; Vazyme, Nanjing, China) was utilized. In 96-well plates, we planted 2×10^3^ cells per well. Then, the plate was incubated for two hours at 37°C in the dark with 10 μl of CCK-8 reagent (A311-01, Vazyme, Nanjing, China) per well. To assess the viability of the cells, the absorbance was measured at 450 nm wavelength using a microplate reader (A33978, Thermo, USA) at 24, 48, 72, and 96 hours.

### 2.17 Transwell assay

Cell invasive potential was assessed using a Matrigel invasion assay (BD Biosciences). Briefly, 20,000 cells in 100 μL serum-free medium were seeded in the upper chamber of transwells. The lower chamber contained 500 μL complete media with 10% FBS. Following a 24-hour incubation at 37°C, invaded cells were fixed, stained with crystal violet, and meticulously removed from the upper chamber. Invaded cells were counted in five random fields under a microscope, and the mean number of invaded cells represented invasion viability.

### 2.18 Colony formation

For colony formation, 500 cells per well were seeded in triplicate 35 mm dishes with a complete growth medium. After incubation for approximately 2 weeks, colonies were fixed with 4% paraformaldehyde for 15 minutes and stained with 0.2% crystal violet for quantification using ImageJ 1.54 (NIH).

### 2.19 Wound healing assay

Cell migration was assessed using a wound healing assay. Cells were seeded in 35 mm dishes with complete medium and allowed to reach 70-90% confluence. A scratch wound was created using a 200 µL pipette tip. The initial wound width (s0) was measured under a microscope. After 24 hours of incubation in a complete medium, the medium was replaced with a serum-free medium, and the wound width (s24) was measured again after an additional 24 hours. Percent wound closure was calculated as [(s0 - s24)/s0] x 100%.

### 2.20 Lentivirus production and transduction

To produce lentivirus particles, HEK293T cells were transfected with the empty vector pLKO.1 containing targeted shRNA sequences for SERPINE1 knockdown, or pLV/EF1A vector containing targeted shRNA sequences for ZFP36 overexpression, along with the helper plasmid pMD2.G. GP-Transfer-Mate was utilized as a transfection reagent for low-scale preparations at a 4:3 ratio of GP-Transfer-Mate to DNA. Moreover, the ratio of the lentiviral backbone constructs pSPAX2 and pMD2.G was 4:3:1. After transfection, the viral supernatant was collected 24 and 48 hours later, spun at 1500 rpm for 5 minutes, flash frozen, and stored at -80°C. MGC803 and HGC27 cells were transduced with lentivirus when reached about 80% confluency, by incubating them in 1.5 mL of media containing 250 μL of lentivirus for 24 hours.

### 2.21 ROS assay

Intracellular ROS levels were assessed using a ROS assay kit (Beyotime, China) according to the manufacturer’s protocol. Briefly, cells were seeded at a density of 10,000 cells/cm² and allowed to adhere. Following treatment, cells were washed twice with PBS and incubated with 10 μM DCFH-DA and 5 μM DAF-FM DA in a serum-free medium for 20 minutes at 37°C in the dark. Cells were then washed three times with PBS to remove unbound dye. Finally, ROS generation was visualized using fluorescence microscopy (Olympus, Tokyo, Japan).

### 2.22 Mitochondrial membrane potential measurement

MMP was assessed using a JC-1 mitochondrial membrane potential assay kit (Solarbio, China) according to the manufacturer’s instructions. Briefly, cells were seeded at 10,000 cells/cm² and allowed to adhere. JC-1 dye was added to the cells, and its accumulation within the mitochondria was dependent on ΔΨm. High ΔΨm promotes JC-1 aggregation in the matrix, emitting red fluorescence. Conversely, low ΔΨm results in JC-1 monomers, emitting green fluorescence. Following staining, cells were analyzed by fluorescence microscopy (Olympus, Tokyo, Japan) for qualitative assessment and flow cytometry for quantitative measurement of MMP.

### 2.23 MitoTracker immunofluorescence staining

The cells with a density of approximately 5000 cells/cm^2^ were plated on 35 mm dishes with coverslips in culture medium and incubated at 37°C. For mitochondrial staining, the culture medium was replaced with a medium containing 100 nM MitoTracker Red (Invitrogen, USA), and cells were incubated with the dye at 37°C for 30 min. Then, followed by three washes with PBS, mitochondria were visualized by a laser confocal scanning microscopy (Leica Microsystems CMS GmbH, Germany).

### 2.24 Statistical analysis

Differential expression analysis of genes was conducted using the Wilcoxon test. Univariate Cox analysis was employed to identify genes with prognostic significance. Kaplan-Meier (K-M) survival curves were constructed and compared using the log-rank test. All statistical analyses were performed using R version 4.2.1 (https://www.r-project.org/) along with appropriate packages. Statistical significance was set at p< 0.05.

## 3 Results

### 3.1 Single-cell RNA sequencing data analysis and HMDRGs identification in STAD cohort

**Figure 1:**
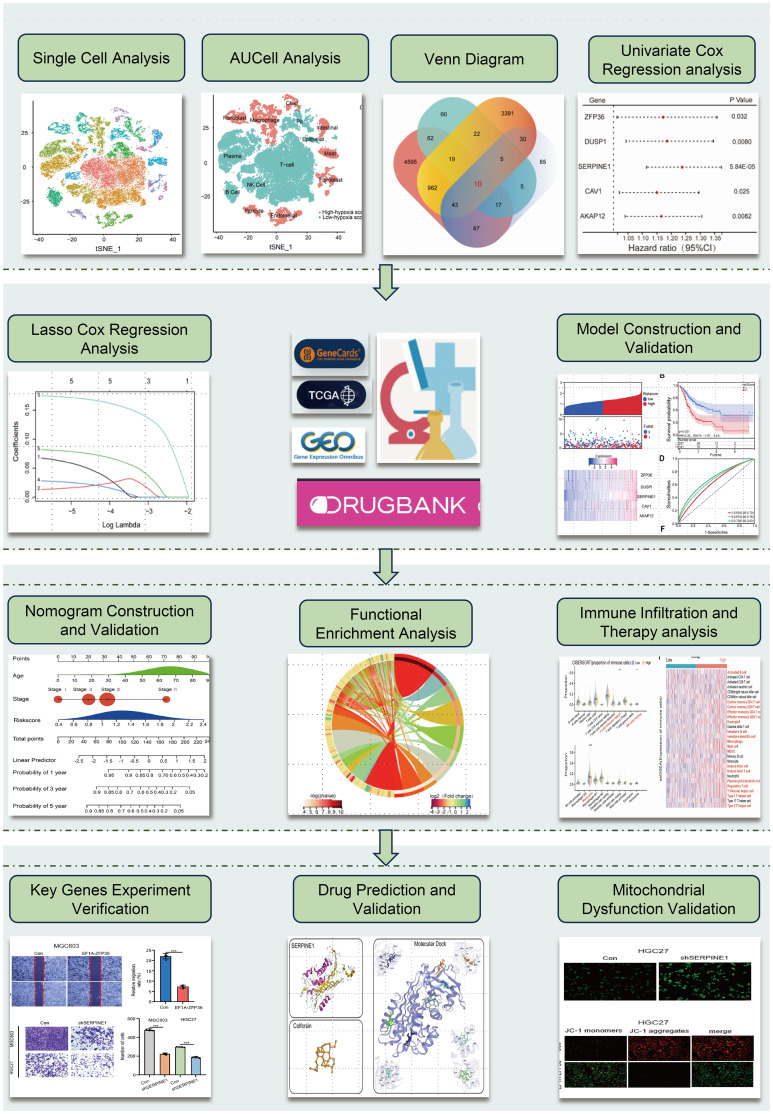
The flowchart of this study.

**Figure 2:**
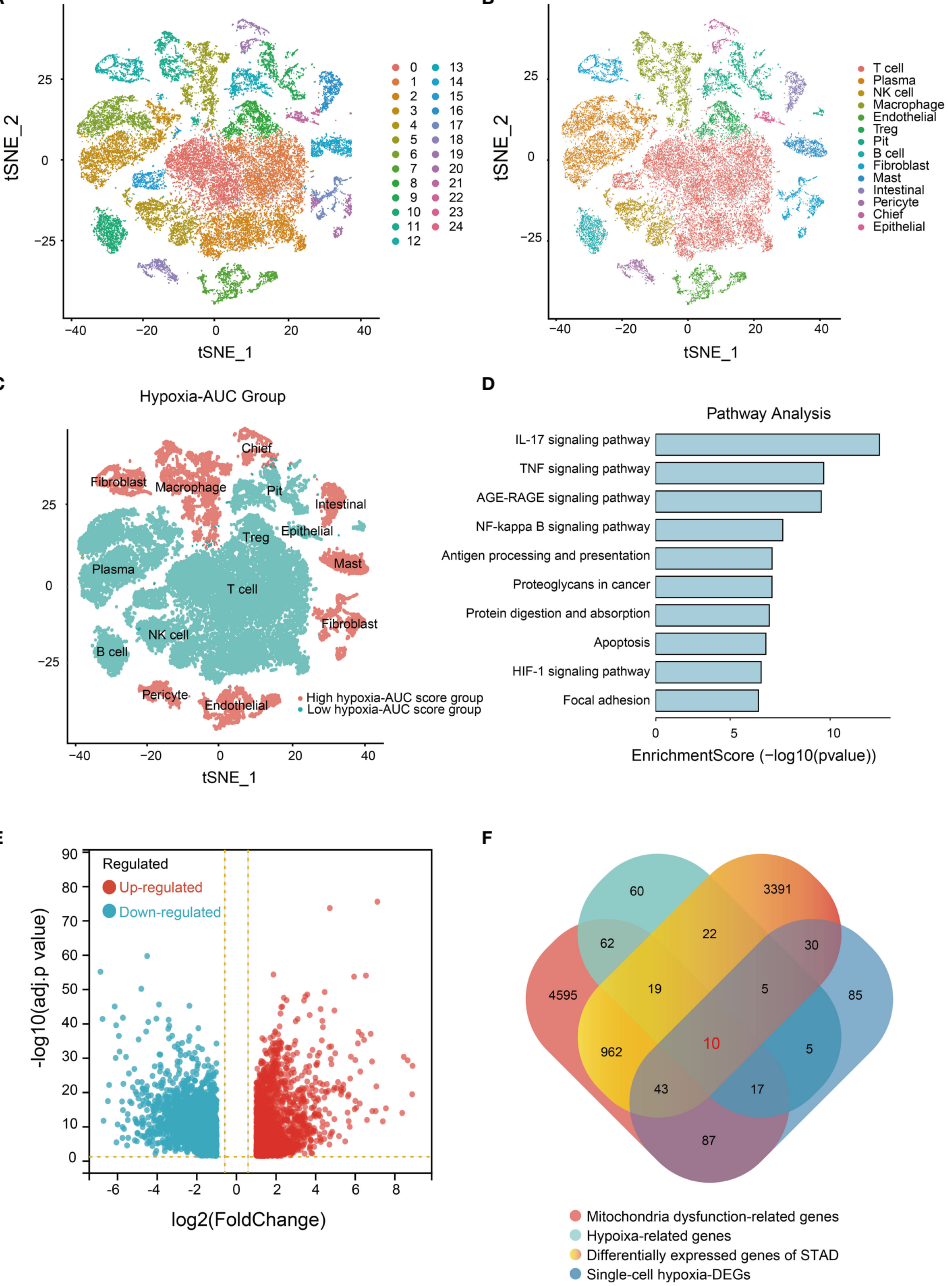
Single-cell RNA sequencing data analysis and HMDRGs identification in STAD cohort. **(A)** The results of the dimension reduction cluster analysis are shown in the t-SNE diagram. **(B)** Cells were annotated into 14 different types of cells. **(C)** All cells were scored according to hypoxia conditions and divided into two groups. **(D)** Enriched pathways analysis of single-cell hypoxia-DEGs. **(E)** Volcano plot of the 4482 DEGs. **(F)** The Venn diagram of analysis of STAD-DEGs, single-cell hypoxia-DEGs, hypoxia-related genes, and mitochondrial dysfunction-related genes.

The flowchart of this study is shown in **Figure 1**. To ensure the integrity and reliability of single-cell transcriptome dataset analysis, we employed quality control by limiting the number of genes detected per cell, each gene expressed at least 3 cells, and the mitochondrial gene ratio (**Supplementary Figure S1A**), a set of 36645 qualified cells were obtained (**Supplementary Figure S1B**). Given the potential variability in cell cycle stages among cells within the dataset, we assessed the distribution of cell cycle phases using cell cycle scoring techniques, revealing a consistent distribution of cell cycle phases across all samples (**Supplementary Figure S1C**). Through a comprehensive examination of clustering outcomes across a spectrum of resolutions ranging from 0.1 to 1.0 utilizing clustree, we found that a resolution of 0.5 yielded reliable results (**Supplementary Figure S1D**). Next, we utilized t-SNE to categorize all cells into 24 detailed clusters (**Figure 2A**). The expression patterns of marker genes were used to identify cell types within these 24 clusters (**Supplementary Figure S1E**), and we identified 14 distinct cell types, including plasma, NK cells, T cells, etc. (**Figure 2B**). Among these 14 different types of cells, to further identify the significantly upregulated- and downregulated- differentially expressed genes (DEGs) under hypoxia conditions from the single-cell level, the AUC score for each cell was calculated by employing the “AUCell” R program. Based on the median AUC scores, 14 cell types were categorized into high- and low-hypoxia AUC groups (**Figure 2C**). 282 single-cell hypoxia-DEGs were found by the “FindMarkers” function with a |log2FoldChange| > 1, adjusted p-value< 0.05, and minimum percentage expression (MinPct) ≥ 0.25 (**Supplementary Table S3**). These DEGs were enriched for pathways associated with IL-17 signaling, TNF signaling, apoptosis, and the HIF-1 signaling pathway (**Figure 2D** and **Supplementary Table S4**). The STAD cohort data comprising 348 patients with detailed clinic parameters, were retrieved from TCGA (**Supplementary Table S5**). The Kaplan-Meier survival curves and log-rank tests for clinicopathological parameters, including overall stage, tumor (T), metastasis (M), and node (N), are shown in **Supplementary Figures S2A-D**, and a total of 4482 STAD differentially expressed genes (STAD-DEGs) exhibited differential expression (**Supplementary Table S6**), of which 2133 genes were up-regulated and 2349 genes were down-regulated as visually represented by the volcano plot (**Figure 2E**). Venn diagram analysis of STAD-DEGs, single-cell hypoxia-DEGs, hypoxia-related genes, and mitochondrial dysfunction-related genes was employed to identify 10 differentially expressed hypoxia- and mitochondrial dysfunction-related genes (HMDRGs) (**Figure 2F**).

### 3.2 Development and evaluation of a prognostic model based on HMDRGs in the TCGA STAD cohort

**Figure 3:**
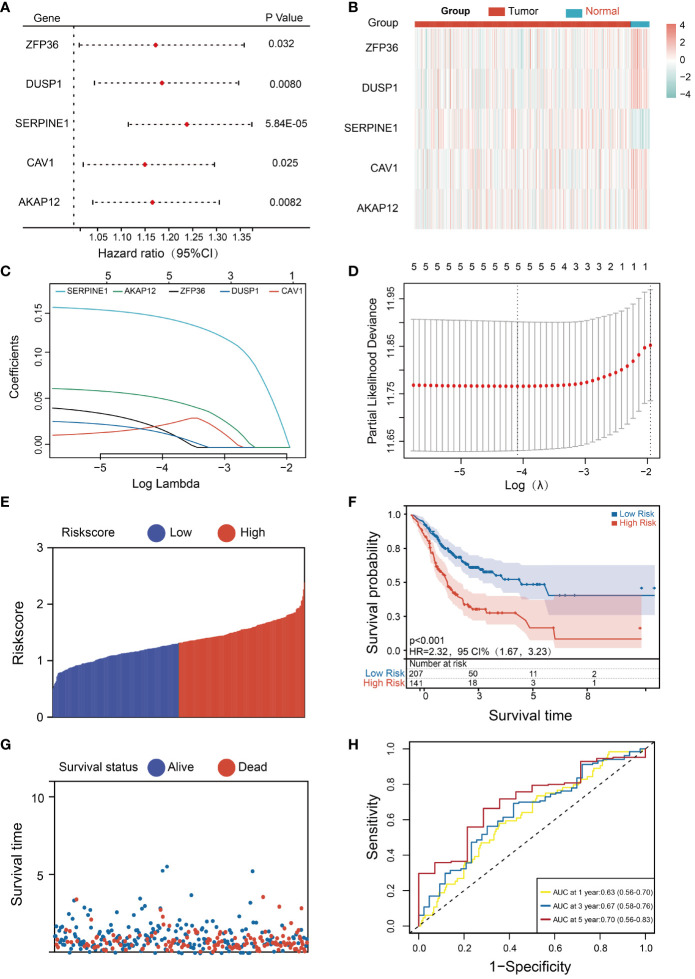
Development and evaluation of the HMDRGs prognostic model. **(A)** Forest plot of the univariate Cox regression analysis. **(B)** Expression levels of survival-related genes in tumor and normal tissues. **(C)** LASSO coefficient profiles of the 5 survival-related genes. **(D)** A coefficient profile plot was produced against the log (lambda) sequence in the LASSO model. The optimal parameter (lambda) was indicated by the black dotted line. **(E, F)** The distributions of the risk score, survival time, and status of patients in TCGA STAD training cohorts. **(G)** Kaplan-Meier curves of the gene signature in TCGA STAD training cohorts. **(H)** The time-dependent ROC curves of the prognostic gene signature in TCGA STAD training cohorts.

To evaluate the prognosis significance of these 10 HMDRGs in the STAD cohort, the univariate Cox regression analysis was performed. Five genes (ZFP36, DUSP1, SERPINE1, CAV1, and AKAP12) were found to be remarkably correlated with OS, and identified as the risk genes (HR>1, p<0.05) (**Figure 3A**). Furthermore, we analyzed the expression levels of these five prognostic genes in normal subjects and STAD patients. The heatmap demonstrated SERPINE1 was up-regulated in tumor tissues, while the rest four genes (ZFP36, DUSP1, CAV1, and AKAP12) were down-regulated as compared to the normal group (**Figure 3B**). We further investigated the expression patterns of these five HMDRGs at the single-cell level, revealing that SERPINE1 exhibited abundant expression in fibroblasts and endothelial cells, ZFP36 showed prominent expression in T cells, NK cells, and B cells, DUSP1 was highly expressed in fibroblasts and macrophages, CAV1 demonstrated remarkable expression in endothelial cells, fibroblasts, and pericytes, and AKAP12 displayed rich expression in fibroblasts, mast cells, and pericytes (**Supplementary Figures S3A-E**). Subsequently, these five HMDRGs underwent LASSO Cox regression analysis to formulate a prognostic risk assessment model in the TCGA training cohort. The LASSO regression analysis of independent HMDRGs confirmed that ZFP36, DUSP1, SERPINE1, CAV1, and AKAP12 were identified as key HMDRGs with an optimal logarithmic lambda value (λ= 0.017) (**Figures 3C, D**). Next, a risk score was assigned to each STAD patient in the TCGA database using the following formula (LASSO Cox regression coefficient * mRNA expression level): Risk score = 0.030 * ZFP36 + 0.024 * DUSP1 + 0.16 * SERPINE1 + 0.016 * CAV1 + 0.068 * AKAP12. According to the median value of the risk scores, we then assessed the prognostic significance of this HMDRG model. Patients in the TCGA training cohort were stratified into low-risk (174 patients) and high-risk (174 patients) groups. Consistently, high-risk patients exhibited higher risk scores (**Figure 3E**) and experienced shorter survival times as compared to low-risk individuals (**Figure 3F**). Moreover, in comparison with the high-risk group, K-M survival analysis demonstrated a higher survival probability in the low-risk group (p<0.001, HR=2.23, 95% CI=1.67-3.23) (**Figure 3G**). The receiver operating characteristic (ROC) analysis indicated that the values for survival probability at 1, 2, and 3 years were 0.63, 0.67, and 0.70, respectively (**Figure 3H**). Importantly, this model was validated in two distinct GEO cohorts (GSE84437 and GSE62254), showing consistent results with those observed in the TCGA training cohort (**Supplementary Figures S4A-H**).

Several prognostic models aimed at predicting survival in STAD patients have been established in previous investigations. Next, we performed a model comparison analysis as compared to other previously reported five models (Model 1 (Li et al.) (42), Model 2 (Deng et al.) (43), Model 3 (Chang et al.) (44), Model 4 (Liu et al.) (45), and Model 5 (Liu et al.) (46)) by using C-Index and decision curve analysis (DCA). A comparative analysis of the C-Index demonstrated that our model (HMDRGs-Model) exhibited the optimal prediction ability for OS probabilities with the highest c-index value (AUC=0.689) compared to the other five models (**Supplementary Figure S5A**). Also, DCA indicated that our model achieved superior performance in clinical practice as evaluated by net benefits (**Supplementary Figure S5B**). These results indicate that the prognostic model based on HMDRGs can offer remarkable accuracy, capability, and performance for clinically predicting the OS of GC patients.

### 3.3 Construction and validation of the nomogram

**Figure 4:**
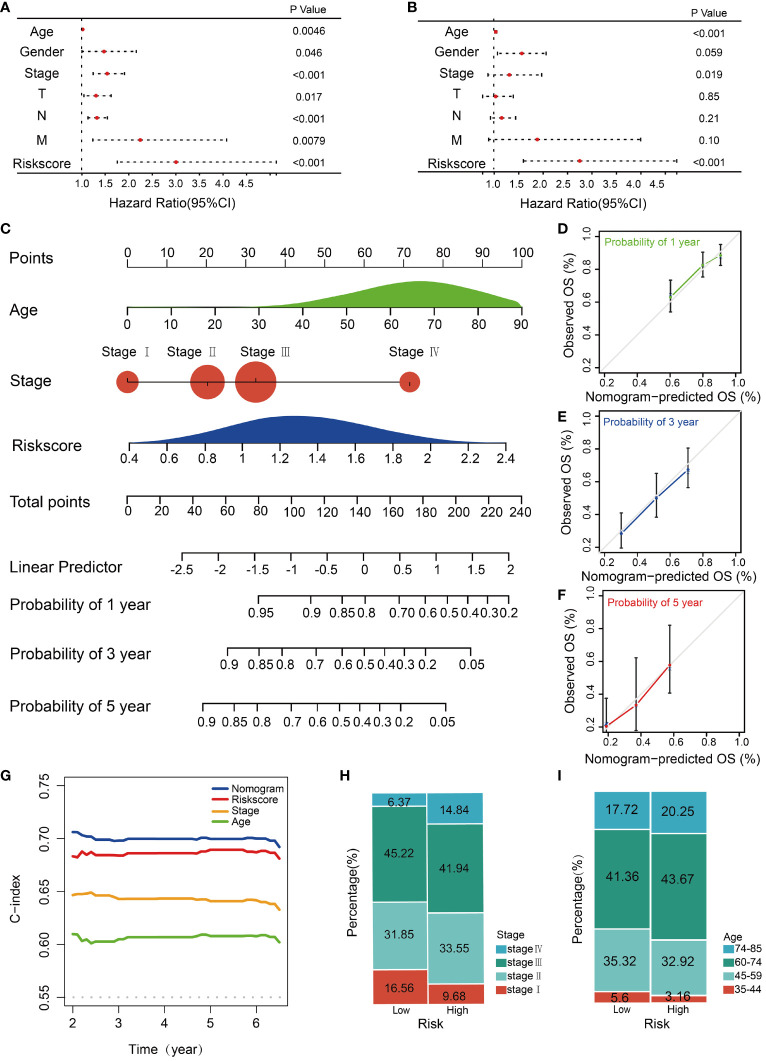
Construction and validation of the nomogram. Forest plots of the **(A)** univariate and **(B)** multivariate Cox regression analysis in TCGA STAD cohorts. **(C)** The nomogram was constructed based on the age, stage and risk score. The calibration plots for the internal validation of the nomogram predicting **(D)** 1-year, **(E)** 3-year, and **(F)** 5- year OS. The X-axis represents the nomogram predicted survival, and the y axis represents the actual survival. **(G)** Concordance index of nomogram, risk score, age, and stage. The percentages of different **(H)** stages and **(I)** ages of patients between high and low-risk groups.

To assess the independent predictive capability of the risk score, univariate and multivariate Cox regression analyses were conducted. The univariate Cox regression analysis revealed a significant association between OS and various clinicopathological parameters, including age (p=0.0046, HR = 1.020, 95% CI =1.0070-1.039), gender (p=0.046, HR=1.47, 95% CI=1.010-2.16), T stage (p=0.017, HR=1.31, 95% CI=1.048-1.63), N stage (p<0.001, HR=1.32, 95% CI=1.13-1.55), M stage (p=0.0078, HR=2.24, 95% CI=1.24-4.08), tumor stage (p<0.001, HR=1.54, 95% CI=1.25-1.92), and risk score (p<0.001, HR=3.00, 95% CI=1.76-5.13) (**Figure 4A**). Additionally, the multivariate Cox regression analysis validated the age (p<0.001, HR = 1.030, 95% CI = 1.012-1.048), tumor stage (p = 0.019, HR = 1.31, 95% CI = 0.87-1.97), and risk score (p<0.001, HR = 2.74, 95% CI = 1.60-4.73) are reliable independent prognostic factors for predicting the OS of STAD patients in the TCGA training cohort (**Figure 4B**). A nomogram serves as an effective tool to integrate multiple risk factors for predicting the OS of cancer patients. Here, we developed a nomogram for predicting 1-year, 3-year, and 5-year OS in the TCGA STAD cohort. Three independent risk factors, including age, stage, and the HMDRG signature, were incorporated into this model (**Figure 4C**). The calibration curves showed that the nomogram-predicted OS aligned with the actual observed OS at 1-year, 3-year, and 5-year intervals (**Figures 4D-F**). The C-Index curve illustrates that the nomogram (AUC=0.702) provides the most accurate prediction compared to other prognostic factors, including risk score (AUC=0.689), tumor stage (AUC=0.646), and age (AUC=0.613) (**Figure 4G**). Besides, we conducted a clinical analysis to discern the variances in clinical features between the two risk groups. Notably, patients in the high-risk group had higher percentages of advanced stages (IV), ages (60-85 years), and TNM classifications (T4, N3, and M1) compared to the low-risk group (**Figures 4H, I** and **Supplementary Figures S6A-C**).

### 3.4 Exploration of molecular functions and signaling pathways related to HMDRGs by GSEA, GO, and KEGG analyses

**Figure 5:**
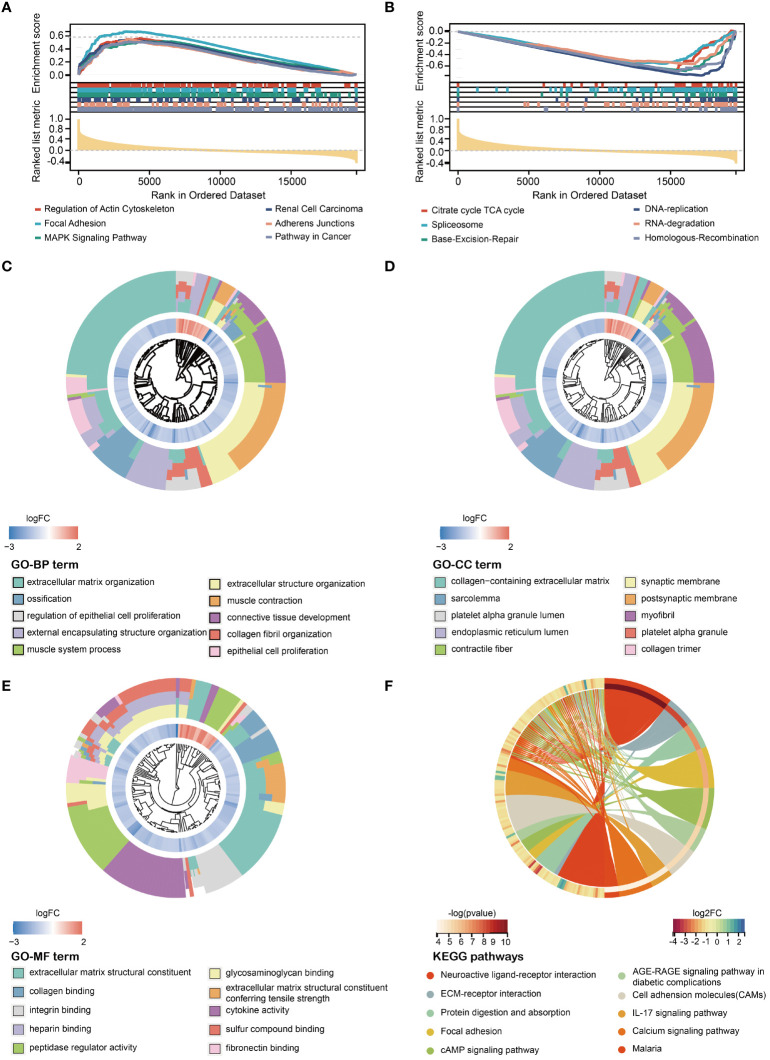
Exploration of molecular functions and signaling pathways of HMDRGs by GSEA, GO, and KEGG analyses. Enrichment plot of the DEGs between the **(A)** high- and **(B)** low-risk groups using GSEA (Gene Set Enrichment Analysis). GO (Gene Ontology) analysis, including **(C)** BP (Biological Process), **(D)** CC (Cellular Component), and **(E)** MF (Molecular Function). **(F)** KEGG (Kyoto Encyclopedia of Genes and Genomes) analysis.

To investigate the underlying differences in biological functions of HMDRGs between the high-risk and low-risk groups, GSEA was employed. All the enriched KEGG pathways were listed in **Supplementary Table S7**. GSEA analysis revealed that 74 pathways exhibited significant enrichment in the high-risk group. Several pathways, including focal adhesion (NES=2.35, p<0.001), regulation of actin cytoskeleton (NES=2.32, p<0.001), mitogen-activated protein kinase (MAPK) signaling pathway (NES=2.19, p<0.001), renal cell carcinoma (NES=2.15, p<0.001), and pathways in cancer (NES=2.14, p<0.001), have been demonstrated to be closely associated tumorigenesis (**Figure 5A**). Nineteen pathways, such as the citrate cycle (TCA cycle) (NES=-2.04, p<0.01), spliceosome (NES=-2.08, p<0.01), base excision repair (NES=-1.97, p<0.001), RNA degradation (NES=-1.93, p<0.01), DNA replication (NES=-1.85, p<0.05), and homologous recombination (NES=-1.84, p<0.01) were significantly enriched in the low-risk group (**Figure 5B**). Next, we explored the differences in biological processes and pathways between the two risk groups based on the HMDRG gene signature. The DEGs between the high-risk group and low-risk group were identified using the cut-off values of adjusted p-values (adj.p)< 0.05 and |log2FoldChange| > 1 (**Supplementary Table S8**). By conducting GO enrichment and KEGG pathway analysis, we identified 722 biological processes (BPs), 106 cellular components (CCs), and 92 molecular functions (MFs) (**Supplementary Table S9**). The top ten enriched BPs, CCs, and MFs are illustrated in **Figures 5C-E**. The KEGG pathway analysis identified 57 enriched pathways (**Supplementary Table S10**), which are significantly enriched in signaling pathways of neuroactive ligand-receptor interaction, extracellular matrix (ECM)-receptor interaction, protein digestion and absorption, focal adhesion, cyclic adenosine monophosphate (cAMP), advanced glycation end product -receptor for AGE (RAGE), cell adhesion molecules (CAMs), IL-17, calcium, and malaria (**Figure 5F**).

### 3.5 Analysis of immune status for STAD patients combined with the prognostic signature

**Figure 6:**
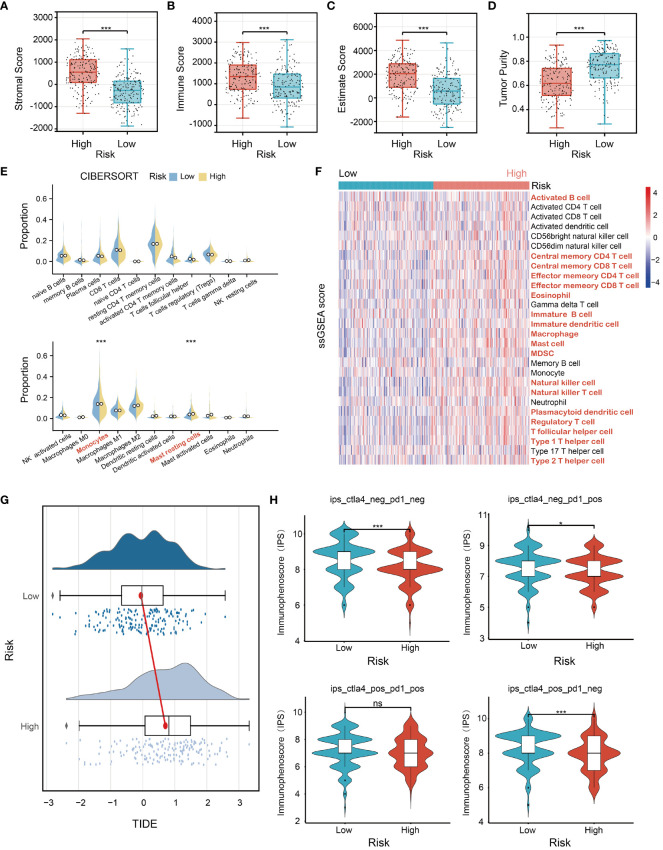
Analysis of immune status for STAD patients combined with the prognostic signature. The comparison analysis of **(A)** stromal scores, **(B)** immune scores, **(C)** ESTIMATE scores, **(D)** tumor purity, **(E)** proportion of immune cells, and **(F)** the ssGSEA (single sample Gene Set Enrichment Analysis) score between the high-risk group and the low-risk group in TCGA STAD cohorts. **(G)** The TIDE (Tumor Immune Dysfunction and Exclusion) analysis of the low- and high-risk groups. **(H)** The IPS (immunophenoscore) analysis of the low- and high-risk groups. *p<0.05, ***p<0.001, ns indicates not significant.

To comprehensively characterize immune cell infiltration within the context of risk stratification in STAD, comprehensive analyses including ESTIMATE, CIBERSORT, and ssGSEA were conducted in high-risk and low-risk groups. ESTIMATE analysis showed that the high-risk group displayed significant elevation in stromal, immune, and estimate scores (p<0.001) (**Figures 6A-C**), while the tumor purity was markedly reduced (p<0.001) (**Figure 6D**). CIBERSORT analysis was performed to evaluate the proportions of 22 immune cell types in the low-risk and high-risk groups, showing that the infiltration levels of the monocyte (p<0.001) and resting mast cells (p<0.001) were remarkably increased in the high-risk group (**Figure 6E**). These findings suggest a positive correlation between the risk score and the infiltration levels of monocytes and resting mast cells in the high-risk group. In addition, the ssGSEA analysis revealed a significant increase in gene expression levels for 18 out of 28 immune cell subtypes in the high-risk group compared to the low-risk group (**Figure 6F**). These findings demonstrate that individuals in the high-risk group tend to have a stronger immunological infiltration than those in the low-risk group. Next, the expression levels of 33 immune checkpoint molecules were examined between the high-risk and low-risk groups. We found that ADORA2A, BTLA, CD200, CD200R1, CD274, CD276, CD28, CD40, CD44, CD48, CD80, CEACAM1, CTLA4, HAVCR2, KIR3DL1, LAG3, LAIR1, NRP1, PDCD1, PDCDLG2, TIGIT, TNSF14, TNFSF18, and TNFSF4 were significantly elevated in the high-risk groups. In contrast, LGALS3 and TNFRSF25 exhibited a significant decrease in the high-risk group (**Supplementary Figure S7**). To further explore the predictive potentials of our model in cancer immunotherapy, we conducted tumor immune dysfunction and exclusion (TIDE) and immunophenoscore (IPS) analyses. The TIDE analysis indicated that the high-risk group with a higher TIDE score presented poorer responses to cancer immunotherapy compared to the low-risk group (**Figure 6G**). In addition, we performed IPS analysis and found that the low-risk group exhibited higher IPS values compared to the high-risk group, suggesting that low-risk patients may be more responsive to immunotherapy using immune checkpoint inhibitors and could achieve better immunotherapeutic efficacy (**Figure 6H**).

### 3.6 *In vivo* validation of HMDRGs expression in GC

To verify the close correlation between the protein expression levels of HMDRGs and the incidence of STAD, we conducted immunohistochemical analysis on both healthy individuals and STAD patients. Consistent with the transcriptional data, immunohistochemistry (IHC) staining retrieved from the Human Protein Atlas (HPA) database revealed that the protein expression levels of AKAP12, CAV1, and ZFP36 were reduced, whereas SERPINE1 was increased in gastric tissue compared to the normal group (**Supplementary Figures S8A-D**). Among them, SERPINE1 and ZFP36 exhibited the most significant changes in staining intensities. These findings provide further *in vivo* evidence to reveal the potential implication of HMDRGs in contributing to STAD pathogenesis and malignant progression.

### 3.7 *In vitro* functional validation of the HMDRGs in gastric cancer cells

**Figure 7:**
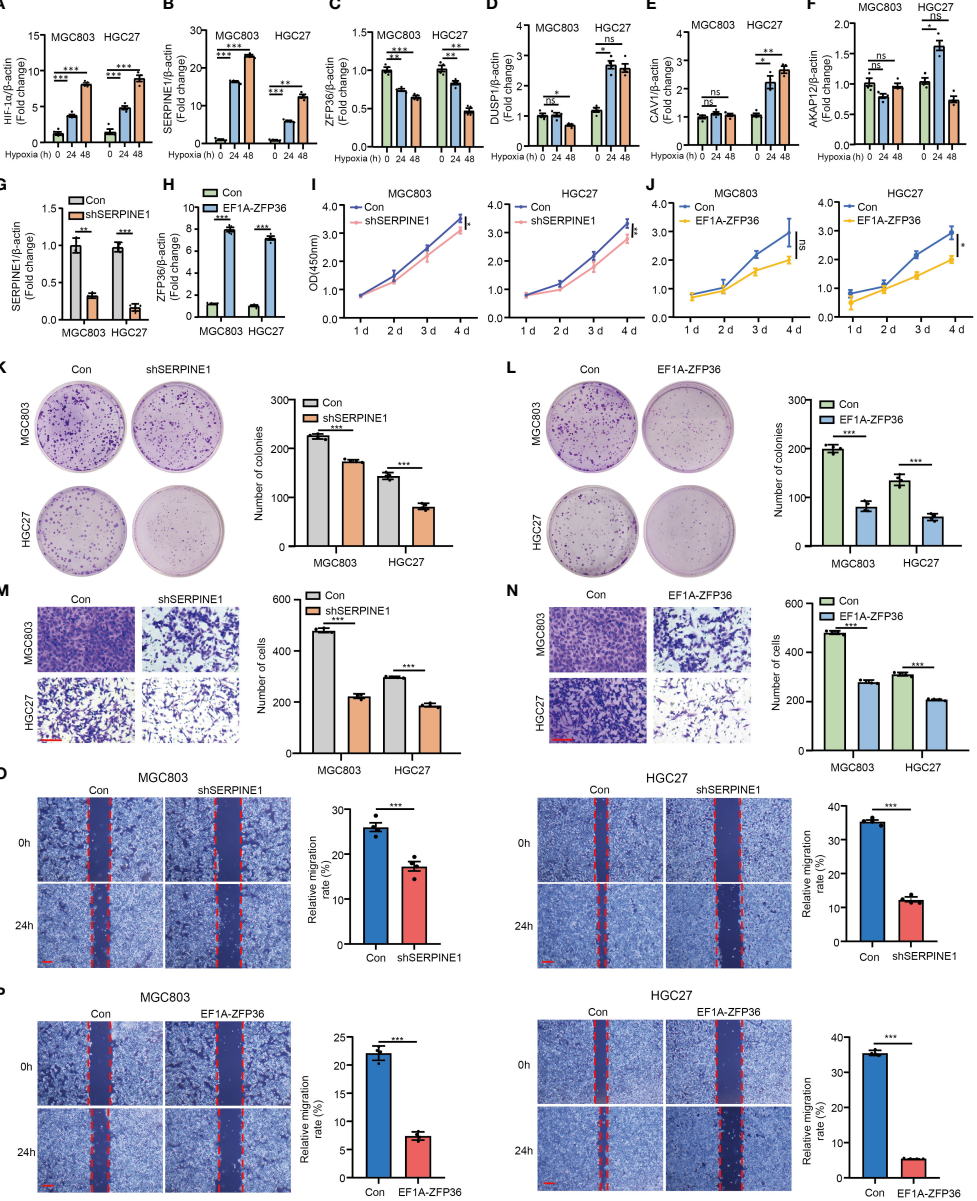
*In vitro* validation of HMDRGs in gastric cancer cells. MGC803 and HGC27 cells were transduced with pLKO.1-TRC shRNA/pLV/EF1A empty vector as control or pLKO.1-SERPINE1 to silence SERPINE1/pLV/EF1A hZFP36 to overexpress ZFP36. RT-qPCR analysis of the mRNA expression of **(A)** HIF-1α, **(B)** SERPINE1, **(C)** ZFP36, **(D)** DUSP1, **(E)** CAV1, and **(F)** AKAP12 under hypoxia conditions in MGC803 and HGC27 cells. RT-qPCR analysis of the mRNA expression of **(G)** the knockdown efficiency of SERPINE1, and **(H)** the overexpression level of ZFP36. **(I, J)** The cell proliferation assay, **(K, L)** colon formation assay, **(M, N)** Transwell assay, and **(O, P)** wound healing assay in MGC803 and HGC27 cells. *p<0.05, **p<0.01, ***p<0.001, ns indicates not significant. Student’s t-test. The error bars represent the mean ± SEM. Scale bar = 0.1 mm.

To explore and validate the hypoxia responses of HMDRGs in gastric cancer cells, MGC803 and HGC27 gastric cancer cell lines were exposed to hypoxic conditions for 0, 24, and 48 hours as verified by the remarkable upregulation of HIF-1α mRNA expression (**Figure 7A**). RT-qPCR analysis demonstrated aberrant expression of five HMDRGs in GC cells under hypoxic conditions. SERPINE1 and ZFP36, exhibiting the most significant upregulation and downregulation, respectively, were selected for further functional validation (**Figures 7B-F**). To further validate the functional role of SERPINE1 and ZFP36 in GC cells, we silenced SERPINE1 and overexpressed ZFP36 in MGC803 and HGC27 cell lines to evaluate the effects on cell viability, proliferation, and migration/invasion. RT-qPCR confirmed the potent silence and overexpression efficiency of SERPINE1 and ZFP36 at the mRNA expression levels, respectively (**Figures 7G, H**). Intriguingly, we observed that silencing SERPINE1 and overexpressing ZFP36 significantly reduced cell viability (**Figures 7I, J**), and suppressed cell proliferation (**Figures 7K, L**), which were respectively evaluated by CCK8 and colony formation assays in both GC cell lines. Transwell assays (**Figures 7M, N**) and cell wound healing (**Figures 7O, P**) demonstrated that SERPINE1 knockdown and ZFP36 overexpression inhibited cell invasion/migration. These *in vitro* results confirm that SERPINE1 functions as a risk gene, while ZFP36 acts as a protective gene in the progression of GC.

**Figure 8:**
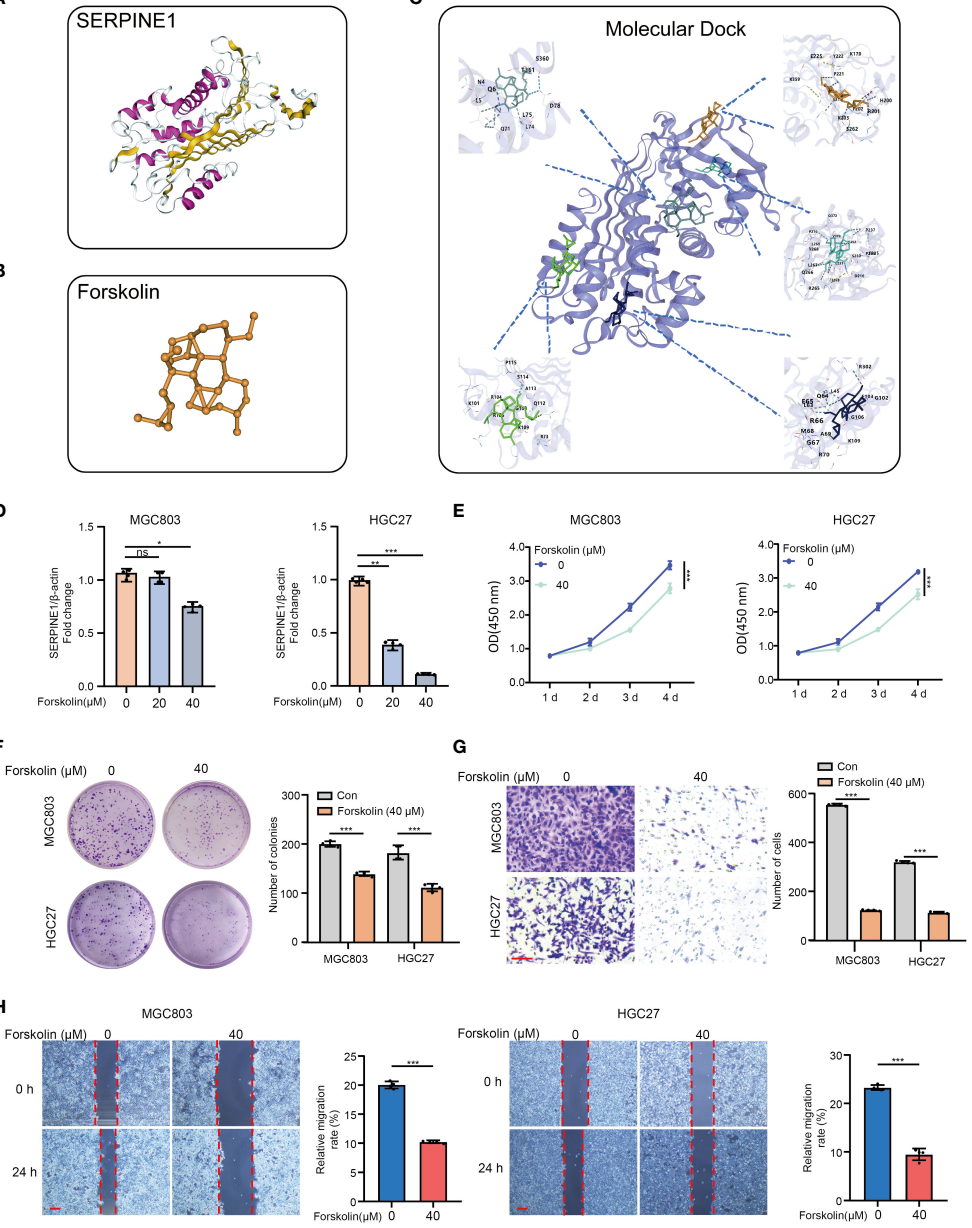
Targeting SERPINE1 by forskolin suppresses the cell proliferation, invasion, and migration in GC cells. **(A)** SERPINE1 molecular structure. **(B)** Forskolin molecular structure. **(C)** Molecular dock between SERPINE1 and forskolin. **(D)** RT-qPCR analysis of the SERPINE1 mRNA expression levels of MGC803 and HGC27 pre-treated with forskolin (0 μM, 20 μM, and 40 μM) for 24h. **(E)** The cell proliferation assay, **(F)** colon formation assay, **(G)** Transwell assay, and **(H)** wound healing assay in MGC803 and HGC27 cells. *p<0.05, **p<0.01, ***p<0.001, ns indicates not significant. Student’s t-test. The error bars represent the mean ± SEM. Scale bar = 0.1 mm.

Furthermore, based on the molecular structure of SERPINE1 (**Figure 8A**), molecular docking analysis demonstrates that forskolin is a potent inhibitor of SERPINE1, exhibiting five binding sites with SERPINE1 (**Figures 8B, C** and **Supplementary Table S11**). As shown in **Figure 8D**, Forskolin (40 μM, 24 h) treatment markedly inhibited the mRNA expression of SERPINE1 in MGC803 and HGC27 cells. Moreover, inhibition of SERPINE1 by forskolin treatment was able to remarkably reduce the GC cell viability (**Figure 8E**), proliferation (**Figure 8F**), and invasion/migration (**Figures 8G, H**), which is consistent with the result that SERPINE1 knockdown alleviated the oncogenic progression of GC cells.

### 3.8 SERPINE1 knockdown and ZFP36 overexpression promote mitochondrial dysfunction in gastric cancer cell

**Figure 9:**
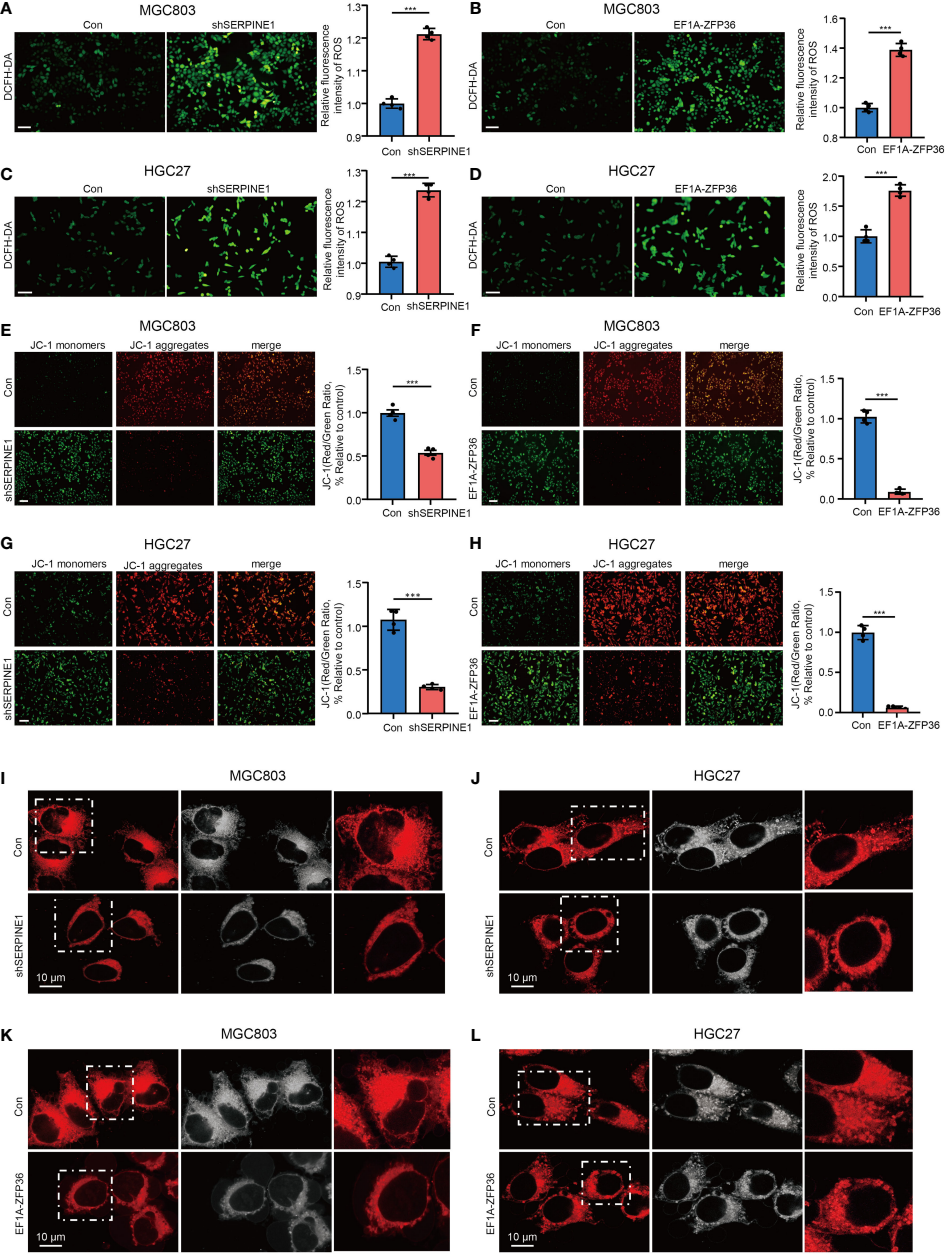
SERPINE1 knockdown and ZFP36 overexpression promote mitochondrial dysfunction in GC cell. MGC803 and HGC27 cells were transduced with pLKO.1-TRC shRNA/pLV/EF1A empty vector as control or pLKO.1-SERPINE1 to silence SERPINE1/pLV/EF1A hZFP36 to overexpress ZFP36. **(A-D)** ROS assay in MGC803 and HGC27 cells. **(E-H)** Mitochondrial membrane potential assay in MGC803 and HGC27 cells. **(I-L)** Confocal microscopy image analysis of the mitochondrial morphology stained with Mitotracker dyes. The red signal indicates mitochondrial distribution. ***p<0.001, Student’s t-test. The error bars represent the mean ± SEM. Scale bar = 0.1 mm.

To further validate whether SERPINE1 and ZFP36 as HMDRGs modulate GC progression is associated with mitochondrial dysfunction, the effects of SERPINE1 knockdown and ZFP36 overexpression on ROS generation and mitochondrial membrane potential were examined in GC cells. DCFH-DA staining analysis demonstrated that silencing SERPINE1 and overexpressing ZFP36 markedly promoted ROS production in GC cell lines MGC803 (**Figures 9A, B**) and HGC27 (**Figures 9C, D**). Moreover, we observed that SERPINE1 knockdown and ZFP36 overexpression resulted in remarkable reductions in mitochondrial membrane potential (**Figures 9E-H**), and mitochondrial morphology damages visualized by the perinuclear clustering of mitochondria as compared to the control groups with more diffused mitochondria (**Figures 9I-L**). These results provided *in vitro* evidence to validate that HMDRGs contribute to GC malignant progression correlated with mitochondrial dysfunction.

## 4 Discussion

GC is a common malignancy characterized by a poor prognosis (47). With the advancement of bioinformatics and single-cell RNA sequencing (scRNA-seq) technology, numerous aberrantly expressed oncogenes have been identified and could serve as prognostic signatures in GC (48, 49). In comparison with previous studies in GC, prognostic gene signatures based on HMDRGs have not been previously investigated. In this study, five HMDRGs were identified and found to be strongly associated with the survival probability of STAD patients via Cox univariate analysis and LASSO regression analysis. A new nomogram that integrates multiple risk factors for predicting the OS of STAD patients stratified clinical outcomes in the TCGA cohorts. Moreover, we provided substantial evidence to validate the reliability and accuracy of the HMDRG signature for the prediction of STAD prognosis and immunotherapy. This study indicates that the HMDRG signature is a convincible biomarker for the prognosis, survival risk stratification, and personalized management of GC. Identification of HMDRGs may provide novel insights for understanding the pathogenesis of GC with a link to hypoxic microenvironment and mitochondrial dysfunction.

Through the bioinformatic analysis of scRNA-seq data of GC, we identified 14 distinct cell types, and found that fibroblasts displayed the highest scores related to hypoxia. The tumor microenvironment comprises a variety of cell types, including fibroblasts, immune cells, nerves, and vascular endothelial cells, all capable of interacting with cancer cells (50). Among these, cancer-associated fibroblasts (CAFs) display the most prevalent constituents. CAFs, one of the most prominent components of the TME, exhibit high sensitivity to hypoxia and engage in crosstalk with cancer cells. Hypoxic CAFs promote cancer malignancy through various mechanisms, including extracellular matrix (ECM) remodeling, immune evasion, metabolic reprogramming, angiogenesis, metastasis, and drug resistance (51). Activated CAFs are capable of producing chemokines, extracellular matrix components, growth factors and metabolites, in turn orchestrating tumor growth through direct contact or paracrine signaling (52, 53). Emerging evidence suggests that CAFs as one of the most abundant mesenchymal cell components of stroma strongly contribute to the initiation and development of GC (54). However, the underlying molecular mechanisms still require further investigation.

In this study, five HMDRGs including ZFP36, SERPINE1, AKAP12, CAV1 and DUSP1, were identified to construct a prognostic risk evaluation model via the LASSO Cox regression analysis, which demonstrates a novel gene signature. In a previous study, a hypoxia-related gene prognostic model was established to guide the drug treatment of GC patients (55). However, here we established HMDRGs prognostic model based on hypoxia- and mitochondrial dysfunction-related genes. Furthermore, based on the mRNA expression levels of these HMDRGs and the coefficients from LASSO Cox regression analysis, a risk score was calculated for each STAD patient. In the TCGA cohort, high-risk patients exhibited elevated risk scores and shorter survival durations compared to low-risk individuals. Kaplan-Meier survival analysis indicated a higher survival probability and longer survival durations in the low-risk group compared to the high-risk group. This model demonstrated consistent results across two independent GEO cohorts. Moreover, we developed a nomogram integrating age, stage, and the HMDRG signature for predicting 1-year, 3-year, and 5-year OS in the TCGA STAD cohorts. Calibration curves of the nomogram demonstrated that the predicted OS closely aligned with the actual observed OS at 1-year, 3-year, and 5-year intervals, suggesting that this nomogram is accurate and reliable for predicting the OS of STAD patients. The prognosis models of GC by bioinformatics have been previously investigated based on telomerase regulation-related lncRNA (56), cuproptosis-related genes (42), ferroptosis-related genes (43, 45, 46), and mitochondrial-related genes (44), whereas our model based on HMDRGs with different approaches present novel advantages. Remarkably, we performed a model comparison analysis with other established prognostic models (42–46), indicating that our model presents superior accuracy and reliability in predicting the OS of GC patients.

To elucidate the potential mechanism of HMDRGs in modulating the malignant processes of GC, we conducted biological function and signaling pathway analyses of HMDRGs in the high-risk and low-risk groups. GSEA enrichment analysis revealed that HMDRGs were involved in regulation of actin cytoskeleton, focal adhesion, and MAPK signaling pathways in the high-risk group. Conversely, in the low-risk group, HMDRGs were enriched in the biological process related to the regulation of the TCA cycle, spliceosome, mismatch repair, and base excision repair. Consistently, a large proportion of HMDRGs-related signaling pathways have been found to be implicated with the orchestration of tumorigenesis. In mesenchymal triple-negative breast cancer (TNBC) cells, hypoxia-induced focal adhesion turnover promotes cell migration (57). Under the hypoxic condition, MAPK signaling cascade enhances HIF-1α stability and transcriptional activity (58). Noteworthy, the MAPK pathways undergo dysregulation through various ways in GC, with their components potentially mediated by trans-regulating factors such as drugs, ligands, or endogenous proteins. Furthermore, within the context of hypoxia, disruption of the TCA cycle occurs in the mitochondria, which results in mitochondrial dysfunction, ultimately contributing to tumor development and metastasis (59). Mechanistically, HMDRGs may contribute to the pathogenesis of GC by regulating hypoxia and mitochondrial dysfunction through the enriched pathways, which offers novel perspectives to elucidate the molecular mechanisms of GC pathogenesis.

The infiltration of immune cells into the tumor microenvironment plays a pivotal role in the development, progression, and malignancy of GC by facilitating interactions with other immune cells (60). To figure out the HMDRGs-related immune cell infiltration in GC, ESTIMATE, CIBERSORT and ssGSEA analyses were performed to examine the involvement of immune cell infiltration in GC in both the high-risk and low-risk groups. In comparison with the low-risk group, GC patients classified in the high-risk group exhibited elevated ESTIMATE score, stromal score, and immune score, alongside a significant decrease in tumor purity. The increase in infiltrating immune cells coincides with stromal activation, potentially hindering the migration of T cells from the tumor parenchyma to the peritumoral stroma (61, 62), in turn leading to an unfavorable prognosis in the high-risk group. In CIBERSORT analysis, we found a significant increase in infiltration levels of monocytes and resting mast cells in the high-risk group evaluated. Furthermore, ssGSEA analysis revealed a significant upregulation in the gene expression levels of immune cells in the high-risk group compared to the low-risk group. These findings lead us to hypothesize that the enhanced presence of infiltrated immune cells in the tumor microenvironment accelerates GC progression, thereby contributing to the poor prognosis observed in the high-risk group. Immune checkpoint inhibitors (ICIs) that suppress the programmed death 1 (PD-1)/programmed death-ligand 1 (PD-L1) and cytotoxic T-lymphocyte antigen 4 (CTLA-4) interactions offer a novel strategy for GC treatment (63). Here, we found that the expression levels of 24 immune checkpoint molecules (ADORA2A, BTLA, CD200, CD200R1, CD274, CD276, CD28, CD40, CD44, CD48, CD80, CEACAM1, CTLA4, HAVCR2, KIR3DL1, LAG3, LAIR1, NRP1, PDCD1, PDCDLG2, TIGIT, TNSF14, TNFSF18, and TNFSF4) were significantly elevated in the high-risk group, while LGALS3 and TNFRSF25 exhibited a significant decrease in the high-risk group. Moreover, immunotherapy response analyses further confirm that the high-risk group with higher TIDE scores and lower IPS exhibit poorer responses to immunotherapy with immune checkpoint inhibitors as compared to the low-risk group, suggesting that our prognostic model offers great potential in clinically predicting immunotherapeutic outcomes for GC immunotherapy.

Remarkably, our experimental work confirms that SERPINE1 and ZFP36 as HMDRGs are indeed hypoxia-related genes, and are also implicated in the regulation of mitochondrial dysfunction in GC cells. Upon hypoxia exposure, SERPINE1 is significantly up-regulated, while ZFP36 is down-regulated in GC cells, which is consistent with previous studies to demonstrate that hypoxia promotes the aberrant expression of SERPINE1 and ZFP36 in cancer cells (64, 65). Importantly, we provide *in vitro* evidence to validate that SERPINE1 depletion and ZFP36 overexpression reduce cell viability/proliferation, and suppress the capability of cell migration/invasion in GC cells, which is in line with the prognostic prediction model based on HMDRGs. Tian et al. demonstrated that SERPINE1 promoted malignant progression and poor prognosis of GC (66), and ZFP36 has been reported to reverse the carcinogenic progression of GC cells (67). Furthermore, forskolin as a potent inhibitor of SERPINE1 has been found, and it is capable of promoting cell death and suppressing cell proliferation and migration/invasion in GC cell lines. Consistently, in GC and non-Hodgkin’s lymphomas, forskolin has been unraveled to exert anticancer by inhibiting cell proliferation and inducing cell apoptosis (68). Nevertheless, whether forskolin provides clinical potential for GC treatment still requires further investigation. Notably, scRNA sequencing analysis reveals that SERPINE1 is prominently expressed in fibroblasts and endothelial cells, while ZFP36 exhibits high expression in T cells and NK cells. Cancer-associated fibroblasts (CAFs) and endothelial cells play significant roles in the microenvironment of GC through ECM remodeling (69), immune modulation (69, 70), and angiogenesis (71, 72). T cells and NK cells can contribute to modulating the immune microenvironment of GC via adaptive immunity (73), and innate immunity (74). Feng et al. have revealed that SERPINE1 can serve as a prognostic biomarker of GC correlated with cuproptosis and can modulate the immune infiltration and angiogenesis in the microenvironment of GC (75). Also, ZFP36 has been identified as a novel senescence-related gene signature for GC prognosis and is involved in regulating the immune microenvironment in patients with GC by influencing the abundance of infiltrating immune cells (76). Although the functional roles of DUSP1, CAV1 and AKAP12 in contributing to GC malignant progression have not been validated in this study, emerging evidence suggests that they may be directly or indirectly involved in the pathogenesis of GC correlating with hypoxia and mitochondrial dysfunction. Recently, DUSP1 has been identified and characterized as a prognostic gene that predicts the overall survival of GC patients (77), and it promotes apatinib-induced resistance for GC therapy by activating the MAPK pathway (78). In hepatocytes, it was reported that hypoxia condition modulated DUSP1 expression in a time-dependent manner (79), and DUSP1 overexpression was able to prevent alcohol-induced mitochondrial dysfunction via reducing ROS production (80). During hypoxic conditions with increased expression of HIF-1α, CAV1 has been demonstrated to be mediated by heat shock protein 90 (HSP90) and acts as a crucial regulator of epithelial-mesenchymal transition (EMT), thereby contributing to GC progression (81). AKAP12, a widely studied tumor suppressor in various cancers, has been demonstrated to be an independent prognostic factor with excellent predictive performance for the prognosis of STAD patients, and its expression is significantly associated with immune cell infiltration, immune pathways, and immunomodulators (82). In human cardiac fibroblasts, reduced AKAP12 expression was reported to contribute to aldosterone-induced mitochondrial dysfunction and cardiac oxidative stress (83). Taken together, this substantial evidence strongly confirms that these HMDRGs (ZFP36, SERPINE1, AKAP12, CAV1, and DUSP1) are strongly involved in hypoxia, mitochondrial dysfunction, and tumor immunity, in turn contributing to the modulation of GC pathogenesis and prognosis.

Although our study established a novel prognostic risk model with remarkable accuracy and reliability based on HMDRGs for GC, our study still bears several limitations. In further investigations, the predictive capacity and reliability of this model for stratifying GC patients, predicting GC prognosis and immunotherapeutic effects need to be validated in practical clinical work using a larger clinical cohort. In addition, the exact molecular mechanism by which HMDRGs contribute to GC pathogenesis and malignant progression still warrants further investigation.

## 5 Conclusion

In summary, we have identified a dependable prognostic HMDRG signature through bioinformatic analysis of hypoxia- and mitochondrial dysfunction-related genes in STAD training cohorts. The prognostic risk model based on HMDRGs demonstrates remarkable reliability and accuracy in stratifying GC patients, predicting GC prognosis, and assessing immunotherapy efficacy, which greatly aids in efficiently managing GC patients and quickly choosing the most effective treatment in clinical practice. Moreover, our study provides new insights into understanding the pathogenesis of GC with a link to hypoxia and mitochondrial dysfunction contributing to the malignant progression and prognosis of GC.

## Data availability statement

Publicly available datasets were analyzed in this study. This data can be found here: HTSeq - Counts (n=407) GDC Hub HTSeq - FPKM (n=407) GDC Hub https://xenabrowser.net/datapages/?cohort=GDC%20TCGA%20Stomach%20Cancer%20(STAD)&removeHub=https%3A%2F%2Fxena.treehouse.gi.ucsc.edu%3A443 HPA https://www.proteinatlas.org/GSE183904, GSE84437 (GPL6947) and GSE62254 (GPL570) https://www.ncbi.nlm.nih.gov/geo/.

## Ethics statement

Ethical approval was not required for the studies on animals in accordance with the local legislation and institutional requirements because only commercially available established cell lines were used.

## Author contributions

YL: Formal Analysis, Validation, Writing – original draft. YC: Formal Analysis, Writing – review & editing. ZW: Writing – review & editing. LW: Writing – review & editing. YY: Project administration, Supervision, Writing – review & editing. YX: Conceptualization, Writing – review & editing.

## References

[1] Sung H Ferlay J Siegel RL Laversanne M Soerjomataram I Jemal A. Global cancer statistics 2020: GLOBOCAN estimates of incidence and mortality worldwide for 36 cancers in 185 countries. CA Cancer J Clin. (2021) 71:209–49. doi: 10.3322/caac.21660 33538338

[2] Machlowska J Baj J Sitarz M Maciejewski R Sitarz R. Gastric cancer: epidemiology, risk factors, classification, genomic characteristics and treatment strategies. Int J Mol Sci. (2020) 21:4012. doi: 10.3390/ijms21114012 32512697 PMC7312039

[3] Lei ZN Teng QX Tian Q Chen W Xie Y Wu K. Signaling pathways and therapeutic interventions in gastric cancer. Signal Transduct Target Ther. (2022) 7:358. doi: 10.1038/s41392-022-01190-w 36209270 PMC9547882

[4] Chen Z Han F Du Y Shi H Zhou W. Hypoxic microenvironment in cancer: molecular mechanisms and therapeutic interventions. Signal Transduct Target Ther. (2023) 8:70. doi: 10.1038/s41392-023-01332-8 36797231 PMC9935926

[5] Bouhamida E Morciano G Perrone M Kahsay AE Della Sala M Wieckowski MR. The interplay of hypoxia signaling on mitochondrial dysfunction and inflammation in cardiovascular diseases and cancer: from molecular mechanisms to therapeutic approaches. Biol (Basel). (2022) 11:300. doi: 10.3390/biology11020300 PMC8869508 35205167

[6] Gilkes DM Semenza GL Wirtz D. Hypoxia and the extracellular matrix: drivers of tumour metastasis. Nat Rev Cancer. (2014) 14:430–9. doi: 10.1038/nrc3726 PMC4283800 24827502

[7] Shida M Kitajima Y Nakamura J Yanagihara K Baba K Wakiyama K. Impaired mitophagy activates mtROS/HIF-1alpha interplay and increases cancer aggressiveness in gastric cancer cells under hypoxia. Int J Oncol. (2016) 48:1379–90. doi: 10.3892/ijo.2016.3359 26820502

[8] Nam S Lee Y. HIF1A protein expression is correlated with clinical features in gastric cancer: an updated systematic review and meta-analysis. Sci Rep. (2024) 14:13736. doi: 10.1038/s41598-024-63019-6 38877062 PMC11178933

[9] Ucaryilmaz Metin C Ozcan G. The HIF-1alpha as a potent inducer of the hallmarks in gastric cancer. Cancers (Basel). (2022) 14:2711. doi: 10.3390/cancers14112711 35681691 PMC9179860

[10] Xiao R Wang S Guo J Liu S Ding A Wang G. Ferroptosis-related gene NOX4, CHAC1 and HIF1A are valid biomarkers for stomach adenocarcinoma. J Cell Mol Med. (2022) 26:1183–93. doi: 10.1111/jcmm.17171 PMC8831942 35023280

[11] Stelzer G Rosen N Plaschkes I Zimmerman S Twik M Fishilevich S. The geneCards suite: from gene data mining to disease genome sequence analyses. Curr Protoc Bioinf. (2016) 54:1 30 1–1 3. doi: 10.1002/cpbi.5 27322403

[12] Fuhrmann DC Brune B. Mitochondrial composition and function under the control of hypoxia. Redox Biol. (2017) 12:208–15. doi: 10.1016/j.redox.2017.02.012 PMC5333533 28259101

[13] Spinelli JB Haigis MC. The multifaceted contributions of mitochondria to cellular metabolism. Nat Cell Biol. (2018) 20:745–54. doi: 10.1038/s41556-018-0124-1 PMC6541229 29950572

[14] Piao HY Liu Y Kang Y Wang Y Meng XY Yang D. Hypoxia associated lncRNA HYPAL promotes proliferation of gastric cancer as ceRNA by sponging miR-431-5p to upregulate CDK14. Gastric Cancer. (2022) 25:44–63. doi: 10.1007/s10120-021-01213-5 34247316

[15] Xia X Wang S Ni B Xing S Cao H Zhang Z. Hypoxic gastric cancer-derived exosomes promote progression and metastasis via MiR-301a-3p/PHD3/HIF-1alpha positive feedback loop. Oncogene. (2020) 39:6231–44. doi: 10.1038/s41388-020-01425-6 32826951

[16] Yang H Hu Y Weng M Liu X Wan P Hu Y. Hypoxia inducible lncRNA-CBSLR modulates ferroptosis through m6A-YTHDF2-dependent modulation of CBS in gastric cancer. J Adv Res. (2022) 37:91–106. doi: 10.1016/j.jare.2021.10.001 35499052 PMC9039740

[17] Lee HC Huang KH Yeh TS Chi CW. Somatic alterations in mitochondrial DNA and mitochondrial dysfunction in gastric cancer progression. World J Gastroenterol. (2014) 20:3950–9. doi: 10.3748/wjg.v20.i14.3950 PMC3983450 24744584

[18] Pei JP Zhang CD Yusupu M Zhang C Dai DQ. Screening and validation of the hypoxia-related signature of evaluating tumor immune microenvironment and predicting prognosis in gastric cancer. Front Immunol. (2021) 12:705511. doi: 10.3389/fimmu.2021.705511 34249015 PMC8267919

[19] Liu J Lichtenberg T Hoadley KA Poisson LM Lazar AJ Cherniack AD. An integrated TCGA pan-cancer clinical data resource to drive high-quality survival outcome analytics. Cell. (2018) 173:400–16.e11. doi: 10.1016/j.cell.2018.02.052 29625055 PMC6066282

[20] Leek JT Johnson WE Parker HS Jaffe AE Storey JD. The sva package for removing batch effects and other unwanted variation in high-throughput experiments. Bioinformatics. (2012) 28:882–3. doi: 10.1093/bioinformatics/bts034 PMC3307112 22257669

[21] Hao Y Stuart T Kowalski MH Choudhary S Hoffman P Hartman A. Dictionary learning for integrative, multimodal and scalable single-cell analysis. Nat Biotechnol. (2024) 42:293–304. doi: 10.1038/s41587-023-01767-y 37231261 PMC10928517

[22] Aran D Looney AP Liu L Wu E Fong V Hsu A. Reference-based analysis of lung single-cell sequencing reveals a transitional profibrotic macrophage. Nat Immunol. (2019) 20:163–72. doi: 10.1038/s41590-018-0276-y PMC6340744 30643263

[23] Aibar S Gonzalez-Blas CB Moerman T Huynh-Thu VA Imrichova H Hulselmans G. SCENIC: single-cell regulatory network inference and clustering. Nat Methods. (2017) 14:1083–6. doi: 10.1038/nmeth.4463 PMC5937676 28991892

[24] Chen Y Lun AT Smyth GK. From reads to genes to pathways: differential expression analysis of RNA-Seq experiments using Rsubread and the edgeR quasi-likelihood pipeline. F1000Res. (2016) 5:1438. doi: 10.12688/f1000research.8987.2 27508061 PMC4934518

[25] Nagashima K Sato Y. Information criteria for Firth's penalized partial likelihood approach in Cox regression models. Stat Med. (2017) 36:3422–36. doi: 10.1002/sim.7368 PMC6084330 28608396

[26] Linden A Yarnold PR. Modeling time-to-event (survival) data using classification tree analysis. J Eval Clin Pract. (2017) 23:1299–308. doi: 10.1111/jep.12779 28670833

[27] Tibshirani R. The lasso method for variable selection in the Cox model. Stat Med. (1997) 16:385–95. doi: 10.1002/(ISSN)1097-0258 9044528

[28] Engebretsen S Bohlin J. Statistical predictions with glmnet. Clin Epigenetics. (2019) 11:123. doi: 10.1186/s13148-019-0730-1 31443682 PMC6708235

[29] Heagerty PJ Lumley T Pepe MS. Time-dependent ROC curves for censored survival data and a diagnostic marker. Biometrics. (2000) 56:337–44. doi: 10.1111/j.0006-341X.2000.00337.x 10877287

[30] Yu G Wang LG Han Y He QY. clusterProfiler: an R package for comparing biological themes among gene clusters. OMICS. (2012) 16:284–7. doi: 10.1089/omi.2011.0118 PMC3339379 22455463

[31] Subramanian A Tamayo P Mootha VK Mukherjee S Ebert BL Gillette MA. Gene set enrichment analysis: a knowledge-based approach for interpreting genome-wide expression profiles. Proc Natl Acad Sci U.S.A. (2005) 102:15545–50. doi: 10.1073/pnas.0506580102 PMC1239896 16199517

[32] The Gene Ontology C. The Gene Ontology Resource: 20 years and still GOing strong. Nucleic Acids Res. (2019) 47:D330–D8. doi: 10.1093/nar/gky1055 PMC6323945 30395331

[33] Kanehisa M Furumichi M Tanabe M Sato Y Morishima K. KEGG: new perspectives on genomes, pathways, diseases and drugs. Nucleic Acids Res. (2017) 45:D353–D61. doi: 10.1093/nar/gkw1092 PMC5210567 27899662

[34] Newman AM Liu CL Green MR Gentles AJ Feng W Xu Y. Robust enumeration of cell subsets from tissue expression profiles. Nat Methods. (2015) 12:453–7. doi: 10.1038/nmeth.3337 PMC4739640 25822800

[35] Hanzelmann S Castelo R Guinney J. GSVA: gene set variation analysis for microarray and RNA-seq data. BMC Bioinf. (2013) 14:7. doi: 10.1186/1471-2105-14-7 PMC3618321 23323831

[36] Bindea G Mlecnik B Tosolini M Kirilovsky A Waldner M Obenauf AC. Spatiotemporal dynamics of intratumoral immune cells reveal the immune landscape in human cancer. Immunity. (2013) 39:782–95. doi: 10.1016/j.immuni.2013.10.003 24138885

[37] Qin Y Liu Y Xiang X Long X Chen Z Huang X. Cuproptosis correlates with immunosuppressive tumor microenvironment based on pan-cancer multiomics and single-cell sequencing analysis. Mol Cancer. (2023) 22:59. doi: 10.1186/s12943-023-01752-8 36959665 PMC10037895

[38] Charoentong P Finotello F Angelova M Mayer C Efremova M Rieder D. Pan-cancer immunogenomic analyses reveal genotype-immunophenotype relationships and predictors of response to checkpoint blockade. Cell Rep. (2017) 18(1):248–62. doi: 10.1016/j.celrep.2016.12.019 28052254

[39] Feng D Zhu W Shi X Wang Z Wei W Wei Q. Immune-related gene index predicts metastasis for prostate cancer patients undergoing radical radiotherapy. Exp Hematol Oncol. (2023) 12:8. doi: 10.1186/s40164-022-00367-x 36635777 PMC9835256

[40] Liu J Shi Y Zhang Y. Multi-omics identification of an immunogenic cell death-related signature for clear cell renal cell carcinoma in the context of 3P medicine and based on a 101-combination machine learning computational framework. EPMA J. (2023) 14:275–305. doi: 10.1007/s13167-023-00327-3 37275552 PMC10236109

[41] Xu Q Chen S Hu Y Huang W. Landscape of immune microenvironment under immune cell infiltration pattern in breast cancer. Front Immunol. (2021) 12:711433. doi: 10.3389/fimmu.2021.711433 34512634 PMC8429934

[42] Li J Kong C Song W Fu T. Identification of cuproptosis-related subtypes, establishment of a prognostic signature and characterization of the tumor microenvironment in gastric cancer. Int J Gen Med. (2023) 16:1631–52. doi: 10.2147/IJGM.S404847 PMC10164657 37168531

[43] Deng H Lin Y Gan F Li B Mou Z Qin X. Prognostic model and immune infiltration of ferroptosis subcluster-related modular genes in gastric cancer. J Oncol. (2022) 2022:5813522. doi: 10.1155/2022/5813522 36276279 PMC9584706

[44] Chang J Wu H Wu J Liu M Zhang W Hu Y. Constructing a novel mitochondrial-related gene signature for evaluating the tumor immune microenvironment and predicting survival in stomach adenocarcinoma. J Transl Med. (2023) 21:191. doi: 10.1186/s12967-023-04033-6 36915111 PMC10012538

[45] Liu SJ Yang YB Zhou JX Lin YJ Pan YL Pan JH. A novel ferroptosis-related gene risk signature for predicting prognosis and immunotherapy response in gastric cancer. Dis Markers. (2021) 2021:2385406. doi: 10.1155/2021/2385406 34868391 PMC8642032

[46] Liu G Ma JY Hu G Jin H. Identification and validation of a novel ferroptosis-related gene model for predicting the prognosis of gastric cancer patients. PloS One. (2021) 16:e0254368. doi: 10.1371/journal.pone.0254368 34252149 PMC8274920

[47] Joshi SS Badgwell BD. Current treatment and recent progress in gastric cancer. CA Cancer J Clin. (2021) 71:264–79. doi: 10.3322/caac.21657 PMC9927927 33592120

[48] Deng C Deng G Chu H Chen S Chen X Li X. Construction of a hypoxia-immune-related prognostic panel based on integrated single-cell and bulk RNA sequencing analyses in gastric cancer. Front Immunol. (2023) 14:1140328. doi: 10.3389/fimmu.2023.1140328 37180146 PMC10169567

[49] Pei S Zhang P Yang L Kang Y Chen H Zhao S. Exploring the role of sphingolipid-related genes in clinical outcomes of breast cancer. Front Immunol. (2023) 14:1116839. doi: 10.3389/fimmu.2023.1116839 36860848 PMC9968761

[50] Oya Y Hayakawa Y Koike K. Tumor microenvironment in gastric cancers. Cancer Sci. (2020) 111:2696–707. doi: 10.1111/cas.14521 PMC7419059 32519436

[51] Kim I Choi S Yoo S Lee M Kim IS. Cancer-associated fibroblasts in the hypoxic tumor microenvironment. Cancers (Basel). (2022) 14:3321. doi: 10.3390/cancers14143321 35884382 PMC9320406

[52] Ozdemir BC Pentcheva-Hoang T Carstens JL Zheng X Wu CC Simpson TR. Depletion of carcinoma-associated fibroblasts and fibrosis induces immunosuppression and accelerates pancreas cancer with reduced survival. Cancer Cell. (2014) 25:719–34. doi: 10.1016/j.ccr.2014.04.005 PMC4180632 24856586

[53] Rhim AD Oberstein PE Thomas DH Mirek ET Palermo CF Sastra SA. Stromal elements act to restrain, rather than support, pancreatic ductal adenocarcinoma. Cancer Cell. (2014) 25:735–47. doi: 10.1016/j.ccr.2014.04.021 PMC4096698 24856585

[54] Sun H Wang X Wang X Xu M Sheng W. The role of cancer-associated fibroblasts in tumorigenesis of gastric cancer. Cell Death Dis. (2022) 13:874. doi: 10.1038/s41419-022-05320-8 36244987 PMC9573863

[55] Tao G Jiao C Wang Y Zhou Q. Comprehensive analysis of hypoxia-related genes for prognosis, immune features, and drugs treatment strategy in gastric cancer using bulk and single-cell RNA-sequencing. Sci Rep. (2022) 12:21739. doi: 10.1038/s41598-022-26395-5 36526698 PMC9758178

[56] Feng J Tang X Song L Zhou Z Jiang Y Huang Y. A telomerase regulation-related lncRNA signature predicts prognosis and immunotherapy response for gastric cancer. J Cancer Res Clin Oncol. (2023) 149:135–46. doi: 10.1007/s00432-022-04456-6 PMC11798325 36333566

[57] Nguyen TMH Lai YS Chen YC Lin TC Nguyen NT Chiu WT. Hypoxia-induced YAP activation and focal adhesion turnover to promote cell migration in mesenchymal TNBC cells. Cancer Med. (2023) 12:9723–37. doi: 10.1002/cam4.5680 PMC10166962 36757143

[58] Hu L Hu J Huang Y Zheng S Yin J Li X. Hypoxia-mediated activation of hypoxia-inducible factor-1alpha in head and neck squamous cell carcinoma: A review. Med (Baltimore). (2023) 102:e32533. doi: 10.1097/MD.0000000000032533 PMC9829281 36607847

[59] Semenza GL. Regulation of metabolism by hypoxia-inducible factor 1. Cold Spring Harb Symp Quant Biol. (2011) 76:347–53. doi: 10.1101/sqb.2011.76.010678 21785006

[60] Wang L Li Z Li Z Ren Y Qian L Yu Y. Identification of A novel gene signature combining ferroptosis- and immunity-related genes for prognostic prediction, immunotherapy and potential therapeutic targets in gastric cancer. J Cancer. (2023) 14:3457–76. doi: 10.7150/jca.87223 PMC10647194 38021154

[61] Salmon H Franciszkiewicz K Damotte D Dieu-Nosjean MC Validire P Trautmann A. Matrix architecture defines the preferential localization and migration of T cells into the stroma of human lung tumors. J Clin Invest. (2012) 122:899–910. doi: 10.1172/JCI45817 22293174 PMC3287213

[62] Song S Shu P. Expression of ferroptosis-related gene correlates with immune microenvironment and predicts prognosis in gastric cancer. Sci Rep. (2022) 12:8785. doi: 10.1038/s41598-022-12800-6 35610340 PMC9129902

[63] Pan S Li K Huang B Huang J Xu H Zhu Z. Efficacy and safety of immune checkpoint inhibitors in gastric cancer: a network meta-analysis of well-designed randomized controlled trials. Ann Transl Med. (2021) 9:290. doi: 10.21037/atm 33708917 PMC7944325

[64] Lyu F Li Y Yan Z He Q Cheng L Zhang P. Identification of ISG15 and ZFP36 as novel hypoxia- and immune-related gene signatures contributing to a new perspective for the treatment of prostate cancer by bioinformatics and experimental verification. J Transl Med. (2022) 20:202. doi: 10.1186/s12967-022-03398-4 35538543 PMC9092714

[65] Zhang L Cao Y Guo X Wang X Han X Kanwore K. Hypoxia-induced ROS aggravate tumor progression through HIF-1alpha-SERPINE1 signaling in glioblastoma. J Zhejiang Univ Sci B. (2023) 24:32–49. doi: 10.1631/jzus.B2200269 36632749 PMC9837376

[66] Tian S Peng P Li J Deng H Zhan N Zeng Z. SERPINH1 regulates EMT and gastric cancer metastasis via the Wnt/beta-catenin signaling pathway. Aging (Albany NY). (2020) 12:3574–93. doi: 10.18632/aging.v12i4 PMC7066881 32091407

[67] Pan Z Yun H Xiao Y Tong F Liu G Zhang G. MiR-934 exacerbates Malignancy of gastric cancer cells by targeting ZFP36. Iran J Public Health. (2023) 52:1720–9. doi: 10.18502/ijph.v52i8.13411 PMC10512137 37744530

[68] Salzillo A Ragone A Spina A Naviglio S Sapio L. Forskolin affects proliferation, migration and Paclitaxel-mediated cytotoxicity in non-small-cell lung cancer cell lines via adenylyl cyclase/cAMP axis. Eur J Cell Biol. (2023) 102:151292. doi: 10.1016/j.ejcb.2023.151292 36736051

[69] Zhang T Li X He Y Wang Y Shen J Wang S. Cancer-associated fibroblasts-derived HAPLN1 promotes tumour invasion through extracellular matrix remodeling in gastric cancer. Gastric Cancer. (2022) 25:346–59. doi: 10.1007/s10120-021-01259-5 PMC8882084 34724589

[70] Yamamoto Y Kasashima H Fukui Y Tsujio G Yashiro M Maeda K. The heterogeneity of cancer-associated fibroblast subpopulations: Their origins, biomarkers, and roles in the tumor microenvironment. Cancer Sci. (2023) 114:16–24. doi: 10.1111/cas.15609 36197901 PMC9807521

[71] Iha K Sato A Tsai HY Sonoda H Watabe S Yoshimura T. Gastric cancer cell-derived exosomal GRP78 enhances angiogenesis upon stimulation of vascular endothelial cells. Curr Issues Mol Biol. (2022) 44:6145–57. doi: 10.3390/cimb44120419 PMC9776843 36547080

[72] Li Y Hu X Lin R Zhou G Zhao L Zhao D. Single-cell landscape reveals active cell subtypes and their interaction in the tumor microenvironment of gastric cancer. Theranostics. (2022) 12:3818–33. doi: 10.7150/thno.71833 PMC9131288 35664061

[73] Wang B Kohli J Demaria M. Senescent cells in cancer therapy: friends or foes? Trends Cancer. (2020) 6:838–57. doi: 10.1016/j.trecan.2020.05.004 32482536

[74] Keshavjee SH Moy RH Reiner SL Ryeom SW Yoon SS. Gastric cancer and the immune system: the key to improving outcomes? Cancers (Basel). (2022) 14:5940. doi: 10.3390/cancers14235940 36497422 PMC9739366

[75] Feng L Li G Li D Duan G Liu J. Cuproptosis-related gene SERPINE1 is a prognostic biomarker and correlated with immune infiltrates in gastric cancer. J Cancer Res Clin Oncol. (2023) 149:10851–65. doi: 10.1007/s00432-023-04900-1 PMC10423162 37318594

[76] Zhang G Dong K Liu J Zhou W. Prognosis and tumor immune microenvironment of patients with gastric cancer by a novel senescence-related signature. Med (Baltimore). (2022) 101:e30927. doi: 10.1097/MD.0000000000030927 PMC9543082 36221394

[77] Yu J Li H Huang C Chen H. Identification and characterization of ferroptosis-related genes in therapy-resistant gastric cancer. Med (Baltimore). (2024) 103:e38193. doi: 10.1097/MD.0000000000038193 PMC11098190 38758860

[78] Teng F Xu Z Chen J Zheng G Zheng G Lv H. DUSP1 induces apatinib resistance by activating the MAPK pathway in gastric cancer. Oncol Rep. (2018) 40:1203–22. doi: 10.3892/or PMC6072387 29956792

[79] Liu C Shi Y Du Y Ning X Liu N Huang D. Dual-specificity phosphatase DUSP1 protects overactivation of hypoxia-inducible factor 1 through inactivating ERK MAPK. Exp Cell Res. (2005) 309:410–8. doi: 10.1016/j.yexcr.2005.06.022 16081065

[80] Li R Dai Z Liu X Wang C Huang J Xin T. Interaction between dual specificity phosphatase-1 and cullin-1 attenuates alcohol-related liver disease by restoring p62-mediated mitophagy. Int J Biol Sci. (2023) 19:1831–45. doi: 10.7150/ijbs.81447 PMC10092755 37063418

[81] Kannan A Krishnan A Ali M Subramaniam S Halagowder D Sivasithamparam ND. Caveolin-1 promotes gastric cancer progression by up-regulating epithelial to mesenchymal transition by crosstalk of signalling mechanisms under hypoxic condition. Eur J Cancer. (2014) 50:204–15. doi: 10.1016/j.ejca.2013.08.016 24070739

[82] Xu Z Xiang L Peng L Gu H Wang Y. Comprehensive analysis of the immune implication of AKAP12 in stomach adenocarcinoma. Comput Math Methods Med. (2022) 2022:3445230. doi: 10.1155/2022/3445230 36148016 PMC9489422

[83] Ibarrola J Sadaba R Martinez-Martinez E Garcia-Pena A Arrieta V Alvarez V. Aldosterone impairs mitochondrial function in human cardiac fibroblasts via A-kinase anchor protein 12. Sci Rep. (2018) 8:6801. doi: 10.1038/s41598-018-25068-6 29717226 PMC5931570

