# Supplementary material for: Development and validation of a hypoxia- and mitochondrial dysfunction- related prognostic model based on integrated single-cell and bulk RNA sequencing analyses in gastric cancer
Source: Front Immunol. 2024 Aug 6;15:1419133. doi: 10.3389/fimmu.2024.1419133 (PMC11333257; doi:10.3389/fimmu.2024.1419133)
Supplement: Supplementary file 2 [file DataSheet_1.zip › Supplementary Figures.docx]

**
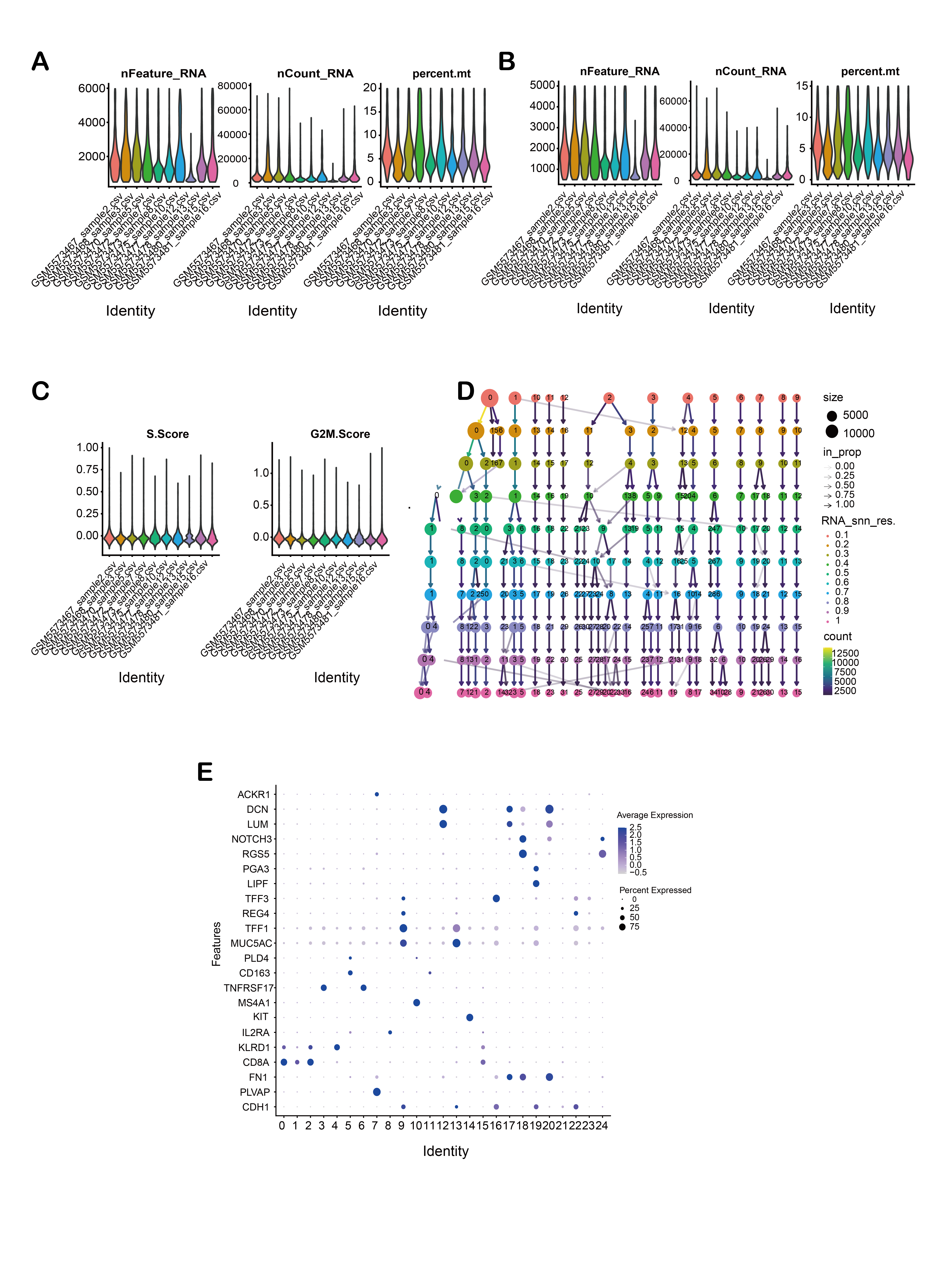
**

**Supplementary Figure S1. The analysis of single-cell sequencing data.** (A) Primary single-cell RNA sequencing data. (B) By limiting the number of genes detected per cell (nFeature_RNA), each gene expressed in cells (nCount_RNA), and the mitochondrial gene ratio (percent.mt), unqualified cells were removed. (C) The cell cycle-related gene expression of all samples. (D) The cell clustering analysis of identified cells. (E) The marker genes expression of identified cells.

**
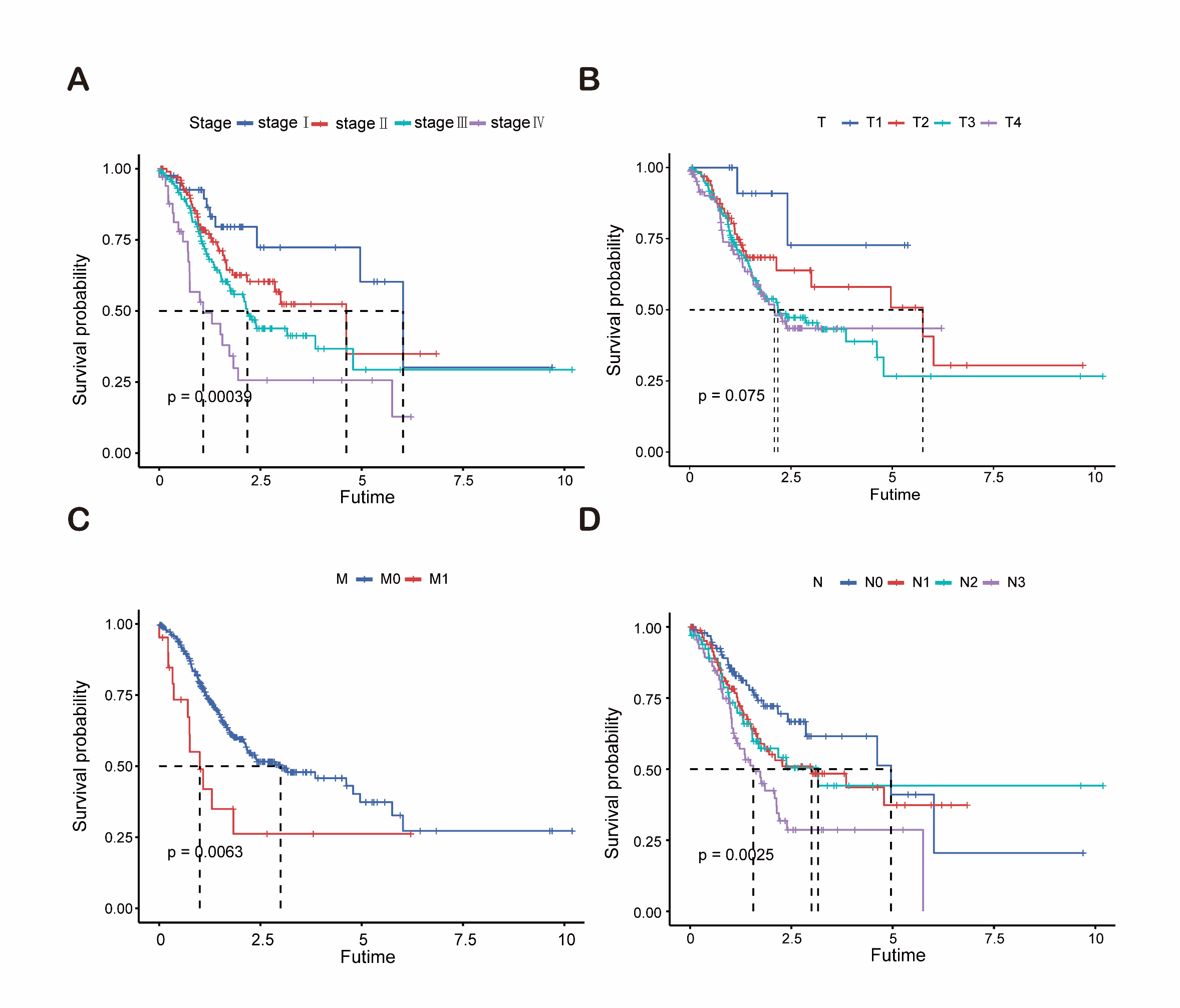
**

**Supplementary Figure S2. The Kaplan-Meier survival curves of TCGA STAD cohort.** (A) Stage I-IV, (B) stage T, (C) stage M, and (D) stage N.

**
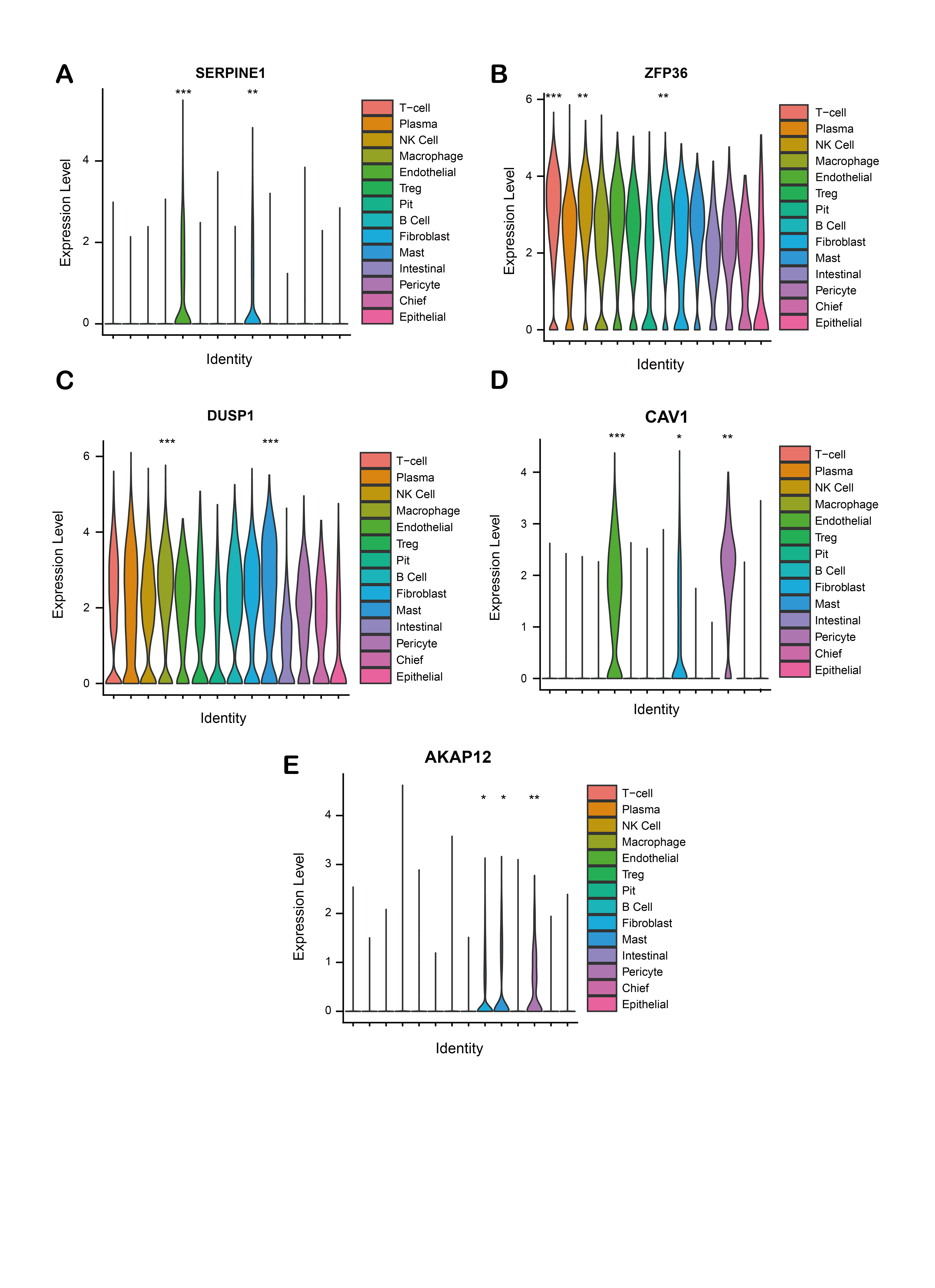
**

**Supplementary Figure S3. The expression profile analysis of five HMDRGs at the single-cell level.** (A) SERPINE1. (B) ZFP36. (C) DUSP1. (D) CAV1. (E) AKAP12. *p<0.05, **p< 0.01, ***p<0.001.

**
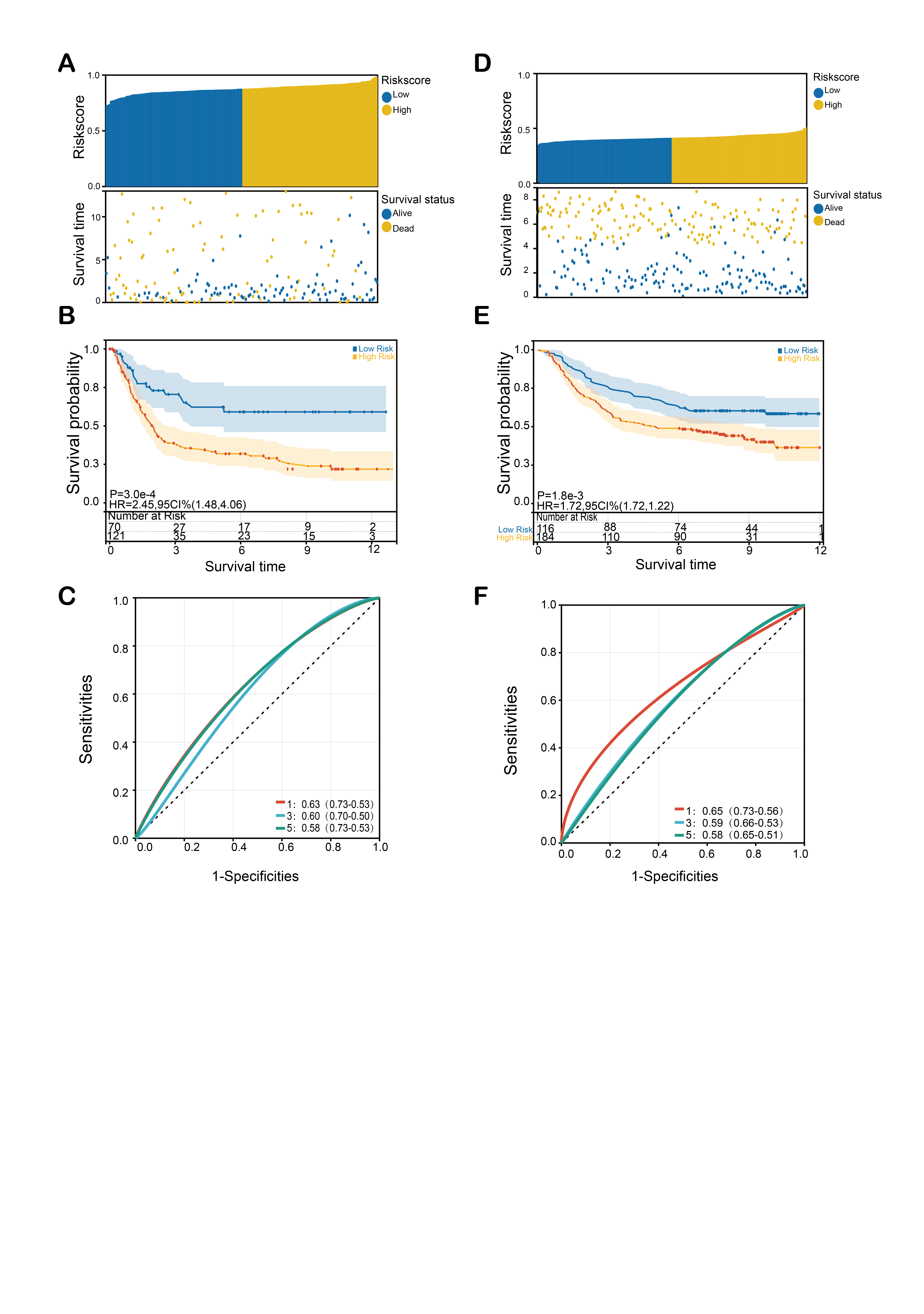
**

**Supplementary Figure S4. Validation of the prognostic HMDRGs model in two GEO cohorts.** (A) The distributions of the risk score, survival time, and status of patients in the GEO84437 validation cohort. (B) Kaplan-Meier curves of the gene signature in the GEO84437 validation cohort. (C) The time-dependent ROC curves of the prognostic gene signature in the GEO84437 validation cohort. (D) The distributions of the risk score, survival time, and status of patients in the GEO62254 validation cohort. (E) Kaplan-Meier curves of the gene signature in the GEO62254 validation cohort. (F) The time-dependent ROC curves of the prognostic gene signature in the GEO62254 validation cohort.

**
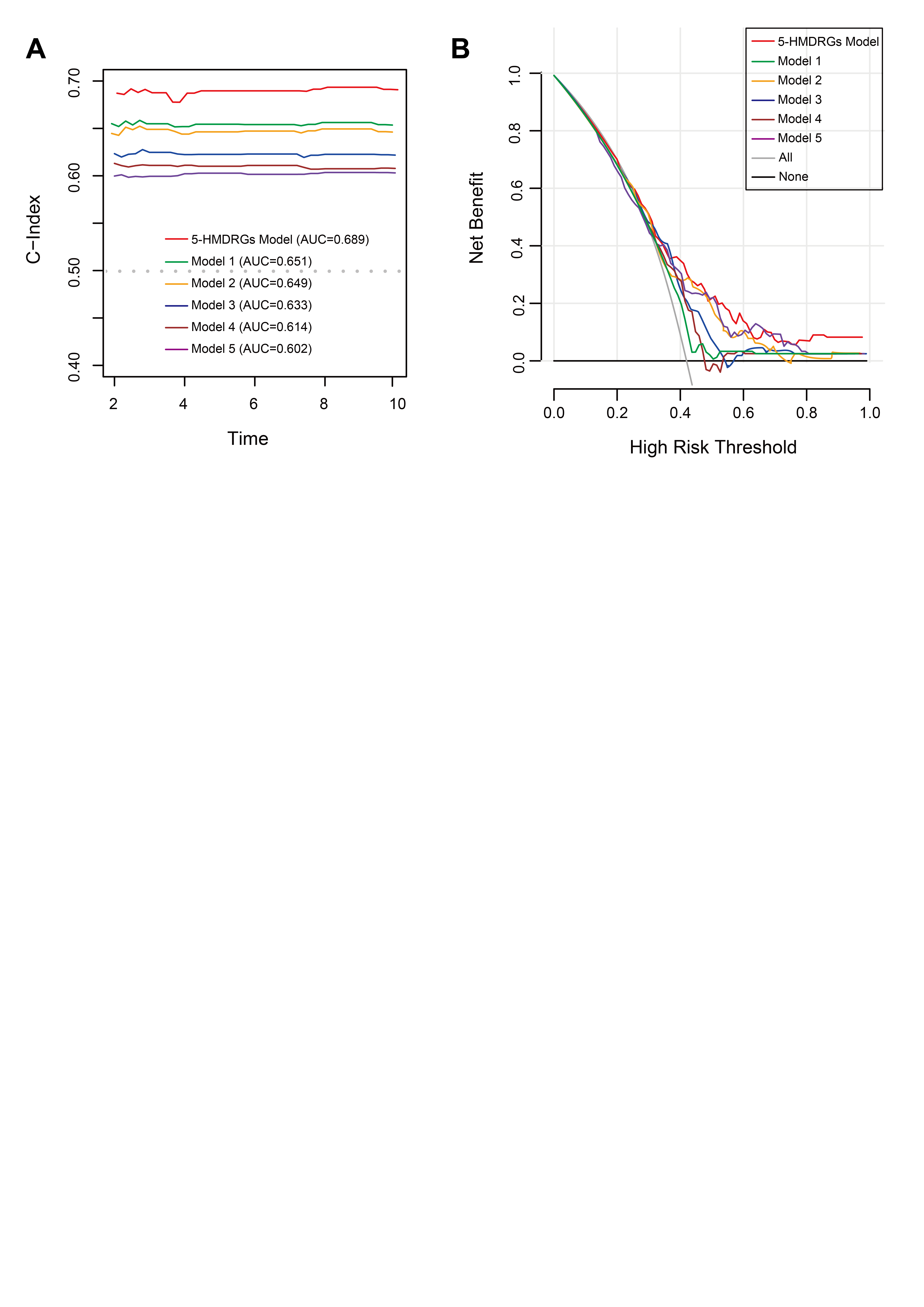
**

**Supplementary Figure S5. The comparison analysis of the HMDRGs model with the other five models.** (A) The C-index curves and (B) DCA profiles analyses of the HMDRGs model and Model 1-5.

**
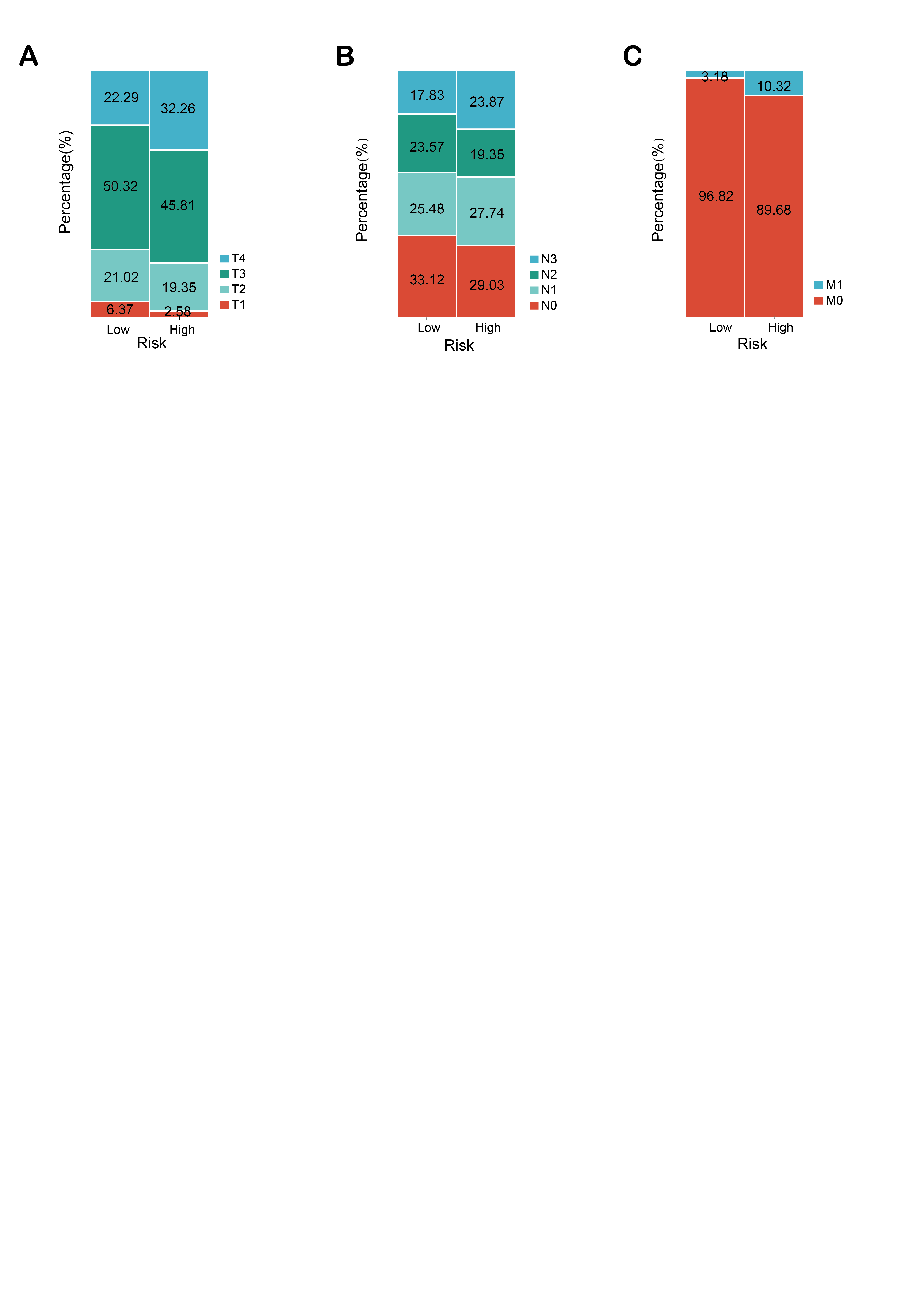
**

**Supplementary Figure S6. The percentages of T, N, and M stage patients between high- and low-risk groups.** The percentages of (A) T stage, (B) N stage, and (C) M stage patients between high and low-risk groups.

**
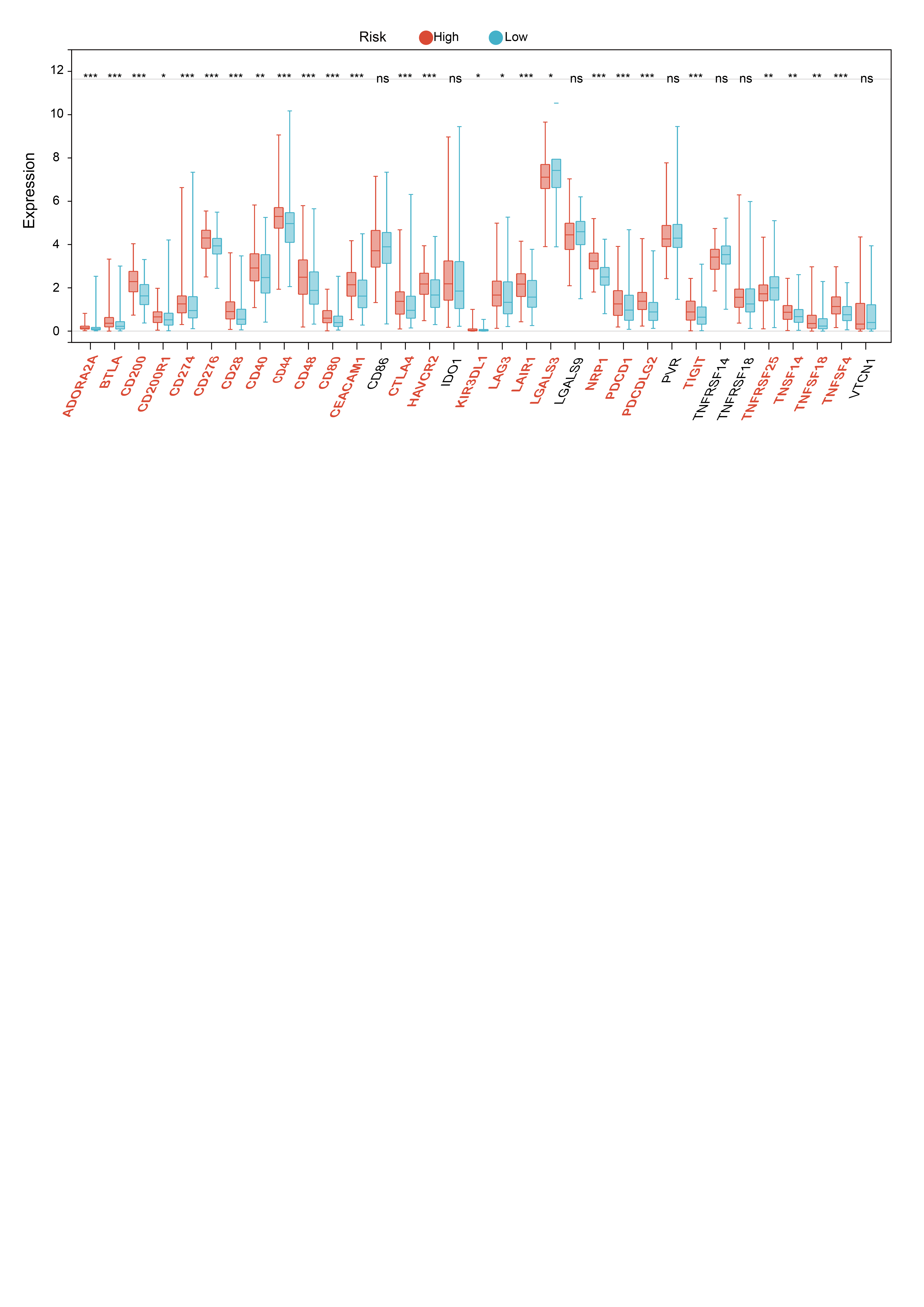
**

**Supplementary Figure S7. Differences in the abundance of immune-checkpoint-related genes between high and low-risk groups.** *p<0.05, **p< 0.01, ***p<0.001, ns indicates not significant.

**
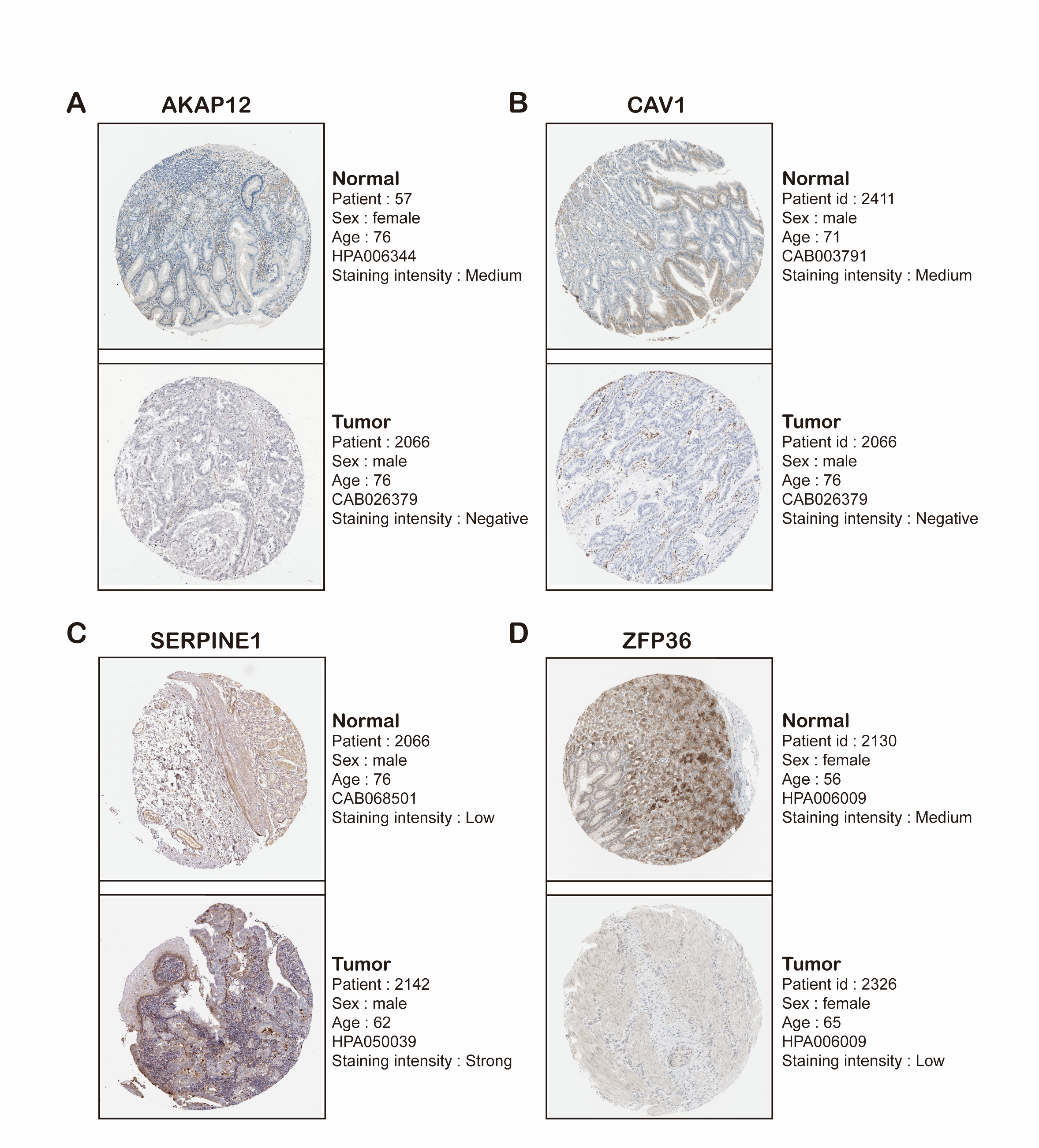
**

**Supplementary Figure S8. Immunohistochemistry staining of the prognostic genes in gastric cancer biopsies**. The representative IHC image of (A) AKAP12 protein, (B) CAV1 protein, (C) SERPINE1 protein, and (D) ZFP36 protein in normal tissues and gastric cancer tissues. The expression frequency and extent of proteins were defined by staining intensity based on the quantification of the percentage of positively stained cells (none cell indicated as “negative”; <25% positive cells indicated as “weak”; 25%-75% positive cells indicated as “moderate”; >75% positive cells indicated as “strong”).
